# Photoswitchable Imines Drive Dynamic Covalent Systems to Nonequilibrium Steady States

**DOI:** 10.1021/jacs.4c03817

**Published:** 2024-07-18

**Authors:** Jiarong Wu, Jake L. Greenfield

**Affiliations:** † Institut für Organische Chemie, Universität Würzburg, Würzburg 97074, Germany; ‡ Center for Nanosystems Chemistry (CNC), Universität Würzburg, Würzburg 97074, Germany

## Abstract

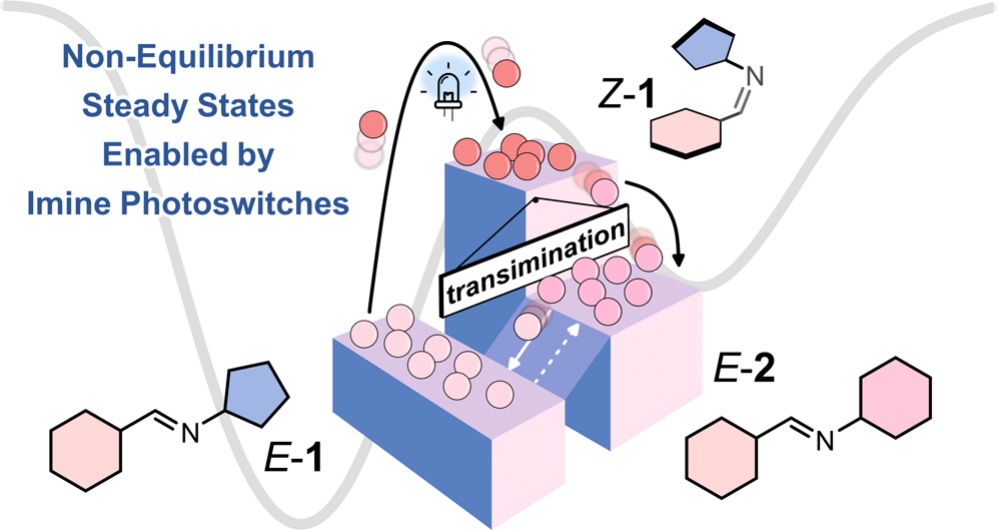

Coupling a photochemical reaction to a thermal exchange process can drive the latter to a nonequilibrium steady state (NESS) under photoirradiation. Typically, systems use separate motifs for photoresponse and equilibrium-related processes. Here, we show that photoswitchable imines can fulfill both roles simultaneously, autonomously driving a dynamic covalent system into a NESS under continuous light irradiation. We demonstrate this using transimination reactions, where *E*-to-*Z* photoisomerism generates a more kinetically labile species. At the NESS, energy is stored both in the metastable *Z*-isomer of the imine and in the system’s nonequilibrium constitution; when the light is switched off, this stored energy is released as the system reverts to its equilibrium state. The system operates autonomously under continuous light irradiation and exhibits characteristics of a light-driven information ratchet. This is enabled by the dual-role of the imine linkage as both the photochromic and dynamic covalent bond. This work highlights the ability and application of these imines to drive systems to NESSs, thus offering a novel approach in the field of systems chemistry.

## Introduction

Generating and maintaining out-of-equilibrium (OOE) states is essential for living systems and in developing complex chemical systems and functional molecular machines.^1−7^ These states rely on a constant supply of energy to preserve the OOE state.^8^ In biological systems, ATP provides this energy,^9^ while artificial systems often use transient chemical additives.^9−11^ This approach, which relies on kinetic asymmetry to overcome microscopic reversibility to reach an OOE state,^12^ often results in the accumulation of byproducts, potentially limiting cyclability. Light, as an alternative energy source, bypasses microscopic reversibility, offers high spatial and temporal control, and limits the production of byproducts, thus facilitating the continuous operation of a process in a closed system.^13^

Photoresponsive units, such as photoswitches^13−16^ and molecular motors,^17−19^ have been employed to shift systems into a new equilibrium state under photoirradiation.^20−24^ Specific examples include light-driven motors that have been used to wind flexible chains into a constrained state^25−29^ and photoswitches that change their geometry to bias the formation of different self-assembled architectures.^20−22,30^ Dynamic covalent systems, particularly those involving imines, have been perturbed to new equilibrium states using various chemical and physical stimuli to induce network responses.^31−35^ Moreover, there has been increasing interest in the photomodulation of an imine bonds’ reactivity toward transimination reactions, offering new possibilities in light-controlled dynamic covalent chemistry.

To date, photomodulation strategies have largely focused on inducing strain at the imine bond, notably in macrocycles^20,21,22^ and molecular cages,^30^ using azo-based photoswitches. In these systems, photoisomerism creates a more conformationally strained state that promotes an otherwise endergonic chemical transformation at the remote dynamic covalent linkage.^20,30^ The complete cycling of these previously reported systems requires the conversion of the metastable *Z*-state back to the *E*-state, achievable either through thermal relaxation, a different wavelength of light, or by using a wavelength of light that affords a ca. 50% photostationary state (PSS) between the *E*/*Z* isomers.^30,36^

Systems that use light to modulate the reactivity of dynamic covalent motifs exhibit similarities to molecular ratchets,^36−39^ particularly energy ratchets.^30,36,40−42^ Energy ratchets require spatial or temporal changes in conditions to perturb the equilibrium state of the system and are thus operated in a stepwise manner.^36,39,43^ In contrast, information ratchets can operate continuously at a nonequilibrium steady state (NESS).^43^ The first example of such an information ratchet was realized experimentally in 2007 by Leigh and coworkers, where a rotaxane with a photoisomerizable axle and a macrocycle functionalized with a photosensitizer could drive the position of the macrocycle on the axle away from thermodynamic equilibrium under photoirradiation.^37^ Building on Leigh’s 2007 information ratchet, Credi and coworkers demonstrated the autonomous driving of an azo-based molecular shuttle into an OOE state under constant light irradiation.^36,44−46^ While examples of dynamic covalent systems exhibiting characteristics of an energy ratchet have been demonstrated,^28,40,42,47,48^ those exhibiting the characteristics of an information ratchet, notably a NESS of the thermal equilibrium process, remain scarce.^28^

It is important to emphasize that while all photochemical isomerization processes operating under continuous illumination result in a NESS due to the photodynamic equilibria,^43^ the critical aspect is the proper coupling of this photochemical process to the chemical network’s thermal exchange process of interest.^37,43^ If successfully achieved, components of the network, including species and steps, that are not directly affected by photoisomerization, can become part of the NESS.^37,41,43^ While the above examples of dynamic covalent systems that can access OOE states under photoirradiation focus on shifting systems to new equilibrium states, designing systems whose thermal exchange processes can achieve NESSs under irradiation remains a challenge.^43^

We have recently introduced a new class of imine-based photoswitches, termed as aryliminopyrazoles (AIPs).^49^ These switches can be prepared quantitatively from commercially available precursors^50^ and display useful photoswitching properties:^13^ achieving over 95% conversion to the metastable *Z*-isomer with visible light, good fatigue resistance, exhibit negative photochromism, and thermal half-lives (*t*_1/2_) of up to 19 h at room temperature. The significant geometric change between the *E*/*Z* isomers of the AIPs and the chemically induced OOE systems of imines explored by Di Stefano and coworkers^51−53^ prompted us to hypothesize whether the AIPs could be coupled with a reversible chemical transformation to drive a thermal exchange process in the system to a NESS under photoirradiation.

**Figure 1:**
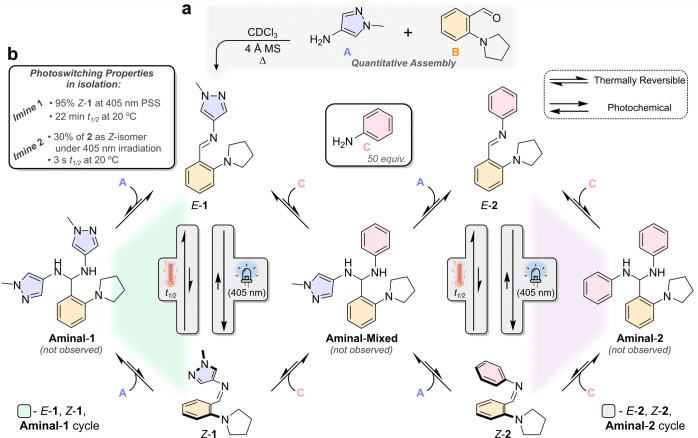
(a) Quantitative assembly of imine *E*-**1** from amine **A** and aldehyde **B** in CDCl_3_. (b) Photoisomerism of *E*-**1** to *Z*-**1** and *E*-**2** to *Z*-**2** was achieved with 405 nm light. The addition of amine **C** to either *E*-**1** or *Z*-**1** induced transimination, producing *E*-**2** and **A**. Gray-shaded boxes indicate unimolecular reaction pathways. The arrows drawn are not to scale. The direction of the arrows is supported by control measurements detailed in Section 5 of the Supporting Information. The aminals were not observed in the ^1^H NMR measurements performed in this study. The *E*-**1**, *Z*-**1**, and **aminal-1** and *E*-**2**, *Z*-**2**, and **aminal-2** cycles are shaded in green and purple, respectively, for clarity.

In this study, we present a system comprising imine-based photoswitches that exhibits characteristics of an autonomously operating, light-driven information ratchet (Figure 1). Under irradiation, the system reaches a NESS, storing energy both in the metastable *Z*-isomer of the imine and in equilibria not directly affected by the photoisomerization process. The NESS of the system’s nonequilibrium constitution is achieved by the photoisomerism of the *E*-imines to their corresponding *Z*-isomers, which undergo transimination at a faster rate. This work highlights the dual functionality of imines as dynamic covalent bonds and photoswitches, demonstrating their potential in light-controlled system chemistry.

## Results and Discussion

Imine *E*-**1** was quantitatively formed from the condensation of **A** and **B** in CDCl_3_ (Figure 1a).^49^ The addition of aniline **C** to *E*-**1** leads to a series of transimination reactions, creating a complex dynamic covalent network as depicted in Figure 1b. The imine components of this network, *E*-**1**, *Z*-**1**, *E*-**2**, and *Z*-**2**, can be accessed either through unimolecular thermal isomerization or photoisomerization pathways (gray boxes, Figure 1b), or through the bimolecular reaction pathways shown. It is important to note that although all the thermal pathways shown are inherently reversible, the equilibria can be strongly biased to one side of the equilibrium reaction, as in the case of the thermal relaxation of *Z*-**1** to *E*-**1**. The photoswitching properties of imines **1** and **2** were investigated in isolation, and control experiments were performed under conditions similar to the network (Figure 1b, Section 3.4 of the Supporting Information).

**Figure 2:**
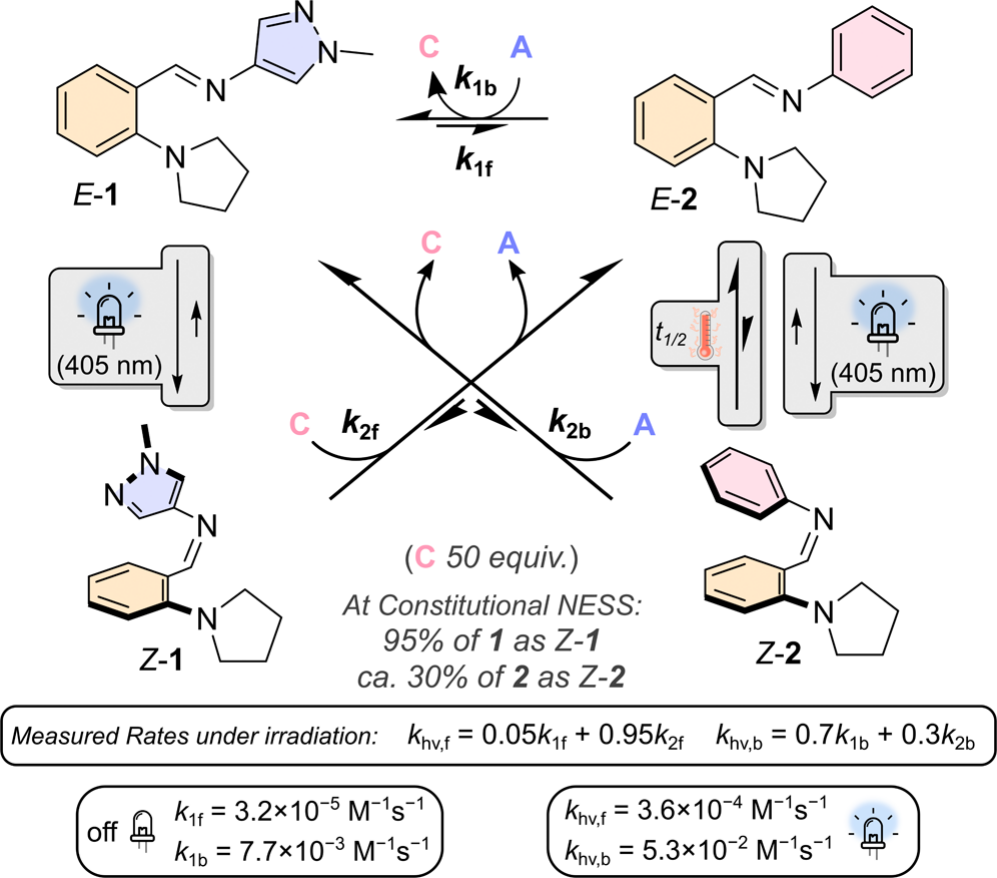
Simplified reaction network of that shown in Figure 1b, highlighting the key transformations probed in ^1^H NMR measurements. The thermal pathway for the conversion of the *E*-isomers to the *Z*-isomers is depicted here, but not observed practically, and thus considered negligible. The reactions shown in the shaded boxes represent unimolecular reactions, while the others are bimolecular. Note that the transimination reactions between **1** and **2** pass through the **aminal-mixed** intermediate, which is not depicted here as the rate constants illustrated are composite values that characterize the rate of conversion between the isomers of imines **1** and **2**. Further discussion supporting this simplified model and control experiments is detailed in Section 5 of the Supporting Information.

**Figure 3:**
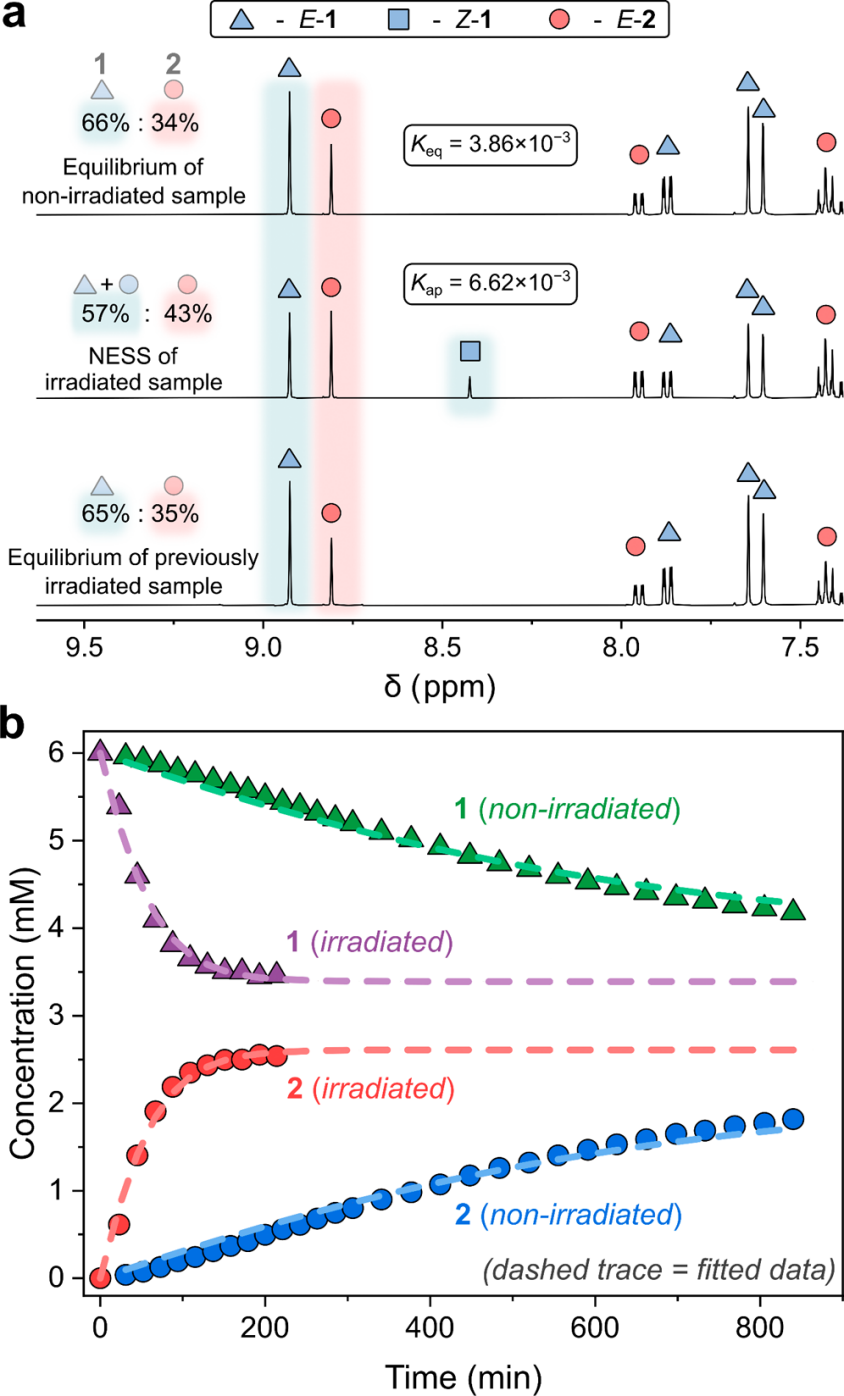
(a) ^1^H NMR spectra (400 MHz, CD_3_CN, 298 K) of a sample consisting of *E*-**1** and **C** (50 equiv): (top) at equilibrium in the dark; (middle) at the NESS achieved with 405 nm irradiation; (bottom) the equilibrium of a previously irradiated sample left in the dark for 18 h. The distribution of the imines, shown as percentages, was determined from the signals shaded in blue and red. Note that the photoisomerism-induced NESS is not directly observed here due to the relaxation of the *Z*-isomers back to the thermodynamically stable *E*-state. (b) The plot of concentration of imines **1** and **2** as a function of time for samples that were either nonirradiated or irradiated. The concentrations were determined from ^1^H NMR kinetic measurements. The fit of the data is shown as dashed traces (Section 4 of the Supporting Information).

The dynamic covalent network shown in Figure 1b can be simplified to its key components; this simplification is justified based on the outcome of control experiments (discussed in Section 5 of the Supporting Information). (i) The *E*-**1**, *Z*-**1**, and **aminal-1** cycles can be omitted (green-shaded cycle, Figure 1b): the 405 nm PSS of **1** remains unchanged upon the addition of **A** (1 equiv.; a 2-fold larger excess of **A** than that generated at the NESS, vide infra), compared to the PSS of **1** measured in isolation. This indicates that the photochemical *E*-**1** to *Z*-**1** pathway is dominant in this part of the cycle. (ii) Given that the 405 nm PSS is obtained for **1**, the impact of the unimolecular thermally induced *Z*-to-*E* isomerization on the amount of *Z*-**1** generated is not significant under continuous photoirradiation.^49^ (iii) The *E*-**2**, *Z*-**2**, and **aminal-2** cycles can also be omitted (purple-shaded cycle, Figure 1b): the amount of *Z*-isomer generated under 405 nm irradiation remains unchanged upon addition of **C** (50 equiv). Thus, in the absence of **A**, the behavior of **2** is determined by the balance of the unimolecular reactions: photoisomerism and the *Z*-to-*E* thermal isomerism processes, affording ca. 30% of **2** as the *Z*-isomer (vide infra). (iv) Given that the formation of *Z*-**1** and *Z*-**2** was not observed from **aminal-1** and **aminal-2**, respectively, we infer that the same is true for the route via **aminal-mixed** (Figures S17 and S28). Therefore, the transimination reactions through **aminal-mixed** yield imines as the *E*-isomer. The control experiments and further discussion of these approximations and simplifications are presented in Section 5 of the Supporting Information. Taking this into account, we propose a simplified network that is more closely related to the observables probed in the experiments, specifically as composite rate constants (Figure 2). Adding aniline (**C**, 50 equiv) to *E*-**1** led to transimination, affording imine *E*-**2** and releasing amine **A**, with the equilibrium favoring *E*-**1** over *E*-**2** (66% *E*-**1** and 34% *E*-**2**). An equilibrium constant *K*_eq_ of 3.86 × 10^–3^ was obtained from ^1^H NMR integration (Figure 3a, Table S4). The higher stability of *E*-**1** compared to *E*-**2** is attributed to the extended conjugation imparted by **A**. It should be noted that 50 equiv of **C** were used to obtain signals of sufficient intensity from the *E*-**2** isomer in the ^1^H NMR spectrum, facilitating analysis and fitting.

We investigated the kinetics of the transimination reaction between *E*-**1** and **C** without photoirradiation using ^1^H NMR spectroscopy. The reaction reached equilibrium after ca. 18 h (Figure 3b). Importantly, the rate constants directly obtained from measurements are composite values that characterize the rate of conversion between isomers of imines **1** and **2** (Figure 2). Analysis of the kinetic data revealed a forward composite rate constant (*k*_1f_) for converting *E*-**1** to *E*-**2** of 3.2 × 10^–5^ M^–1^ s^–1^ and a reverse composite rate constant (*k*_1b_) for converting *E*-**2** to *E*-**1** of 7.7 × 10^–3^ M^–1^ s^–1^ (Figure 2, Supporting Information Section 4). The kinetic data of this nonirradiated sample exhibit a sigmoidal character, indicative of an autocatalytic process. This phenomenon has been previously observed in transimination reactions by Di Stefano and coworkers,^54^ where it was attributed to an increased concentration of base, resulting from the release of aniline during imine hydrolysis. In our study, however, hydrolysis was not observed. We hypothesize that this autocatalytic feature could originate from the liberation of amine **A** as the system approaches equilibrium. The exact mechanism for transimination remains a subject of interest.^54−57^ Nevertheless, only minor deviations are observed between the fit of our simplified model to the experimental data (Figure 3).

Irradiating the system with 405 nm light accelerated the transimination rate, reaching a steady state in ca. 3 h. ^1^H NMR showed conversion of a portion of *E*-**1** to *Z*-**1**, while *Z*-**2** was not observed. It is important to note that *Z*-**2** is generated under 405 nm irradiation but is not observed due to the experimental setup: the short thermal half-life of *Z*-**2** (*t*_1/2_ of 3 s at 20 °C) and the low degree of photoconversion to the *Z*-isomer result in the population of *Z*-**2** thermally isomerizing back to *E*-**2** before the ^1^H NMR measurements. Control measurements using UV/vis spectroscopy indicated that similar proportions of the metastable *Z*-isomers were generated under continuous 405 nm irradiation in the chemical system compared to the photoswitches in isolation. Specifically, 95% of **1** as the *Z*-isomer and ca. 30% of **2** as the *Z*-isomer were achieved for concentrations of imines **1** and **2** at the NESS shown in Figure 3a (Section 3.4 of the Supporting Information). The steady state obtained under irradiation is characterized by an apparent equilibrium constant (*K*_ap_) of 6.62 × 10^–3^. This value is 50% higher than the *K*_eq_ of the nonirradiated sample, indicating a greater population of the less thermodynamically favored isomer, *E*-**2**, under light irradiation. The original thermodynamic equilibrium, as displayed by a nonirradiated sample, was recovered by leaving the sample in the dark for 18 h (Figure 3a), indicating the system’s full reversibility and metastable character.

Following the kinetics of transimination under light irradiation, the rate of forming *E*-**2** increased by a factor of 11.3, with an apparent composite rate constant (*k*_*hv,*f_) of 3.6 × 10^–4^ M^–1^ s^–1^. We infer that the increased rate of forming *E*-**2** is due to the generation of a more kinetically labile, and thermodynamically less stable, species upon photoirradiation, specifically *Z*-**1** (Figure S26). The apparent composite rate constant of the reverse reaction (*k*_*hv,*b_), from *E*-**2** back to *E*-**1**, also increased, but by a smaller factor of 7.0. This increase in the reverse reaction is attributed to the generation of a small population of *Z*-**2**, which is also more reactive toward transimination and is described by the apparent composite rate constant *k*_2b_ (Section 4.2 of the Supporting Information). The extent of this reaction involving *Z*-**2** is limited by the poor photoswitching properties, notably the low amount of *Z*-**2** (ca. 30% of **2**) generated under 405 nm irradiation (Section 3.4 of the Supporting Information). As a control experiment, a sample of *E*-**1** was irradiated to the 405 nm PSS, then, the light was removed, and **C** (50 equiv) was immediately added. The degree of transimination, monitored by ^1^H NMR for the time elapsed since the addition of **C**, corroborated that the presence of *Z*-**1** increases the rate of transimination (Figure S26). Given that no *Z*-**2** was generated under these conditions, we infer that the generation of *Z*-**1** is crucial to achieve the OOE system. As continuous irradiation was not applied in this control measurement and an increased rate in forming *E*-**2** was observed, the possibility of the NESS mechanism occurring from photoinduced SET is unlikely.^58^ Further supporting this, these measurements were performed under an ambient atmosphere; no detectable differences in behavior were observed when the system was operated under a nitrogen atmosphere.

Taking the steady state and kinetics data together, the system’s behavior under photoirradiation indicates that a NESS of the system’s thermal exchange process, linked to the system’s constitution, has been achieved. Specifically, the light-induced change in the apparent composite rate constants and the difference between *K*_eq_ and *K*_ap_ show that the equilibrium between imines **1** and **2** is indeed coupled to the photoswitching event.^43^

The system shown in Figure 2 consists of two linked cycles. The cycle of *E*-**1** to *Z*-**1**, to *E*-**2**, and back to *E*-**1** exhibits an overall anticlockwise flux. Alternatively, Figure 2 can be represented as two triangular cycles forming a diamond, where the overall flux would be described as a net conversion of imine **1** to **2** (Figure S33). We assume that the composite rate constant for the transformation of *E*-**2** to *Z*-**1** is negligible, resulting in unidirectional cycling. However, since *E*-**2** can also photoisomerize to *Z*-**2**, another cycle exists within the system. This second cycle, involving *E*-**2** to *Z*-**2**, to *E*-**1**, and back to *E*-**2**, displays a clockwise flux (or an overall conversion of imine **2** to **1** when represented as two triangular cycles).

Focusing on the anticlockwise cycle (ACWC), light induces the photoisomerism of imine **1**, affording a *Z*-rich NESS of **1**. The energy stored in the metastable *Z*-**1** state is released during the subsequent transimination reaction.^59^ Such photochemical processes inherently impart directionality, originating from a “power stroke” to a lower energy state (an energetically downhill stochastic exchange process).^59−61^ For the three component cycles shown here, the photochemical excitation to an intermediate (*Z*-isomers) and the relaxation to a subsequent intermediate (transimination) and back to the starting state will always be directional, provided that one of the three components in the cycle undergoes photoexcitation.^30,36,43,59^ The system operates continuously and also autonomously under constant photoirradiation, resembling an information ratchet mechanism.

Considering the ACWC and the clockwise cycle (CWC) together, the difference in flux between the two cycles affords the NESS of the imine constitution and is strongly influenced by the amount of *Z*-isomer produced under photoirradiation. Notably, imines **1** and **2** undergo thermal *Z*-to-*E* isomerization at different rates; *Z*-**1** converts slowly enough to allow a 95% *Z*-rich PSS to be achieved under irradiation, whereas *Z*-**2** converts more rapidly, preventing the attainment of a true PSS (30% of **2** as *Z*-isomer at 20 °C). Consequently, the imines display distinctly different *Z*-isomer populations under 405 nm irradiation, thereby establishing a preferred directional bias in the system.

A semiquantitative analysis of the net flux in the system is possible (Section 5.6 of the Supporting Information).^36^ The ratcheting constant *K*_r_ is defined by the composite rate constants of the cycle proceeding in one direction over those acting in the opposite direction when under irradiation. Given that the rate of the thermal bimolecular transimination reaction of *E*-to-*Z* is negligible compared to the rest of the cycle, *K*_r_ becomes large for each cycle and tends toward unidirectionality. As these cycles operate in different directions, but are inherently linked, the quotient of the *K*_r_’s of the two cycles compares the relative flux of the two cycles in the overall system. Taking the case where the ratio of the thermal bimolecular transimination reaction of *E*-to-*Z* for the CWC and ACWC is equal as an example, the flux of the ACWC is 6.1 × 10^3^ times larger than that of the CWC. We propose that this ratio could be a useful figure of merit for comparing other imine-based light-driven ratchets in the future.

From a qualitative viewpoint, while Figure 2 provides a simplified view of the system that aligns closely with experimental observables, it is important to recall that all the transimination reactions proceed through the same **aminal-mixed** intermediate (Figure 1b). The rate constants for the conversion from **aminal-mixed** to either *E*-**1** or *E*-**2** remain the same, regardless of whether the system is in the dark or under photoirradiation. However, the net rate of forming **aminal-mixed** differs greatly depending on the use of light: under irradiation, *Z*-imines also react to form **aminal-mixed**. Focusing on the ACWC cycle, the faster net rate of forming **aminal-mixed** via the *Z*-isomer perturbs the equilibrium between *E*-**1** and *E*-**2**. A portion of **aminal-mixed** (ca. 40%, Figure S32) converts to *E*-**2**. The remaining **aminal-mixed** returns to *E*-**1** and undergoes the same process, leading to the consumption of *E*-**1**, which is balanced by the conversion of *E*-**2** back to **aminal-mixed**. Since the CWC is also operating in the system, the same process occurs in the opposite direction, albeit with a lesser amount of *E*-**2** being converted to *Z*-**2** under photoirradiation. Thus, the relative flux of the two linked cycles, the individual amount of the *Z*-isomer, and their net rates to forming **aminal-mixed** define the system. An overview of the rate constants associated with the overall system shown in Figure 1b is presented in Section 5.5 of the Supporting Information. This photoswitchable dynamic covalent system stands out for its ability to function autonomously under photoirradiation.^36^ Unlike previous systems where the photochromic motif and dynamic covalent bond were spatially separate,^20,28,30^ this system combines both functions within the same imine bond. This approach causes the transimination reaction to reset the metastable *Z*-state back to the *E*-state as the system passes through the **aminal-mixed** intermediate.

**Figure 4:**
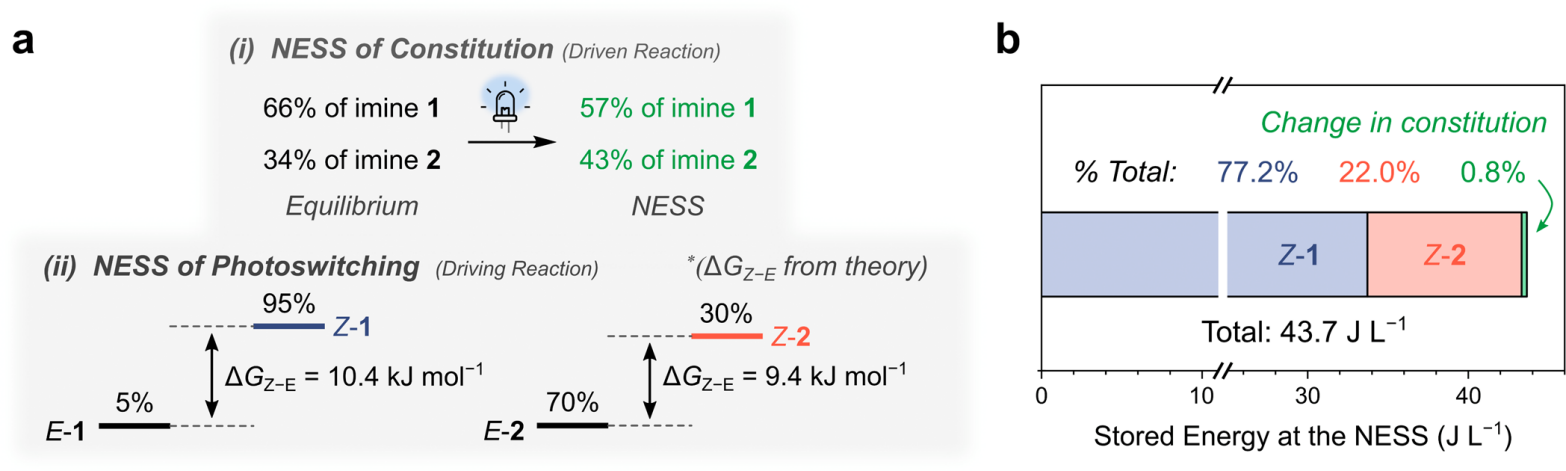
(a) Estimate of the energy stored in the NESS, considering (i) the NESS of the system’s nonequilibrium constitution (driven reaction) and (ii) the inherent NESS achieved by photoisomerism (driving reaction). The Δ*G*_*Z*–*E*_ was calculated from theory (Section 7 of the Supporting Information). (b) Graphical representation of the total energy stored in the NESS and the percentage breakdown of the different contributions (Section 6 of the Supporting Information). Note that this sample is composed of an initial 6 mM solution of imine **1** prepared to a volume of 0.5 mL.

Finally, the free energy stored at the NESS was calculated.^62^ Since photoswitching inherently produces a NESS, the overall system includes contributions from photoisomerization of the *E*-isomers to the metastable *Z*-state and from the change in the concentration of imines **1** and **2** relative to the equilibrium state (Figure 4a).^36^ Given that up to 95% of **1** and 30% of **2** exist as the *Z*-isomer at the NESS (Section 3.4 of the Supporting Information), the energy stored from this photodynamic equilibrium is readily determined from the Δ*G*_*Z–E*_ obtained from quantum chemical calculations (Section 7 of the Supporting Information).^49^ To determine the energy stored in the constitution, specifically the nonequilibrium concentration of the compounds in the system, the change in the relative concentration of the components of the system between the NESS and equilibrium state was calculated.^36^ In total, the system stores 43.7 J L^–1^ of energy at the NESS (Figure 4b). The majority, 99.2%, of this stored energy originates from the *E*/*Z* photoisomerism, with approximately 0.8% of the energy being stored in the NESS constitution (Section 6 of the Supporting Information). Considering only the perturbed thermodynamic equilibrium (i.e., the change in constitution at the NESS), the stored energy of 0.33 J L^–1^ is of similar magnitude to other systems reported in the literature.^36^

## Conclusions

In conclusion, we have demonstrated the application of photoswitchable imines as both light-responsive and dynamic covalent linkages simultaneously. Utilizing these imines, we have designed systems capable of undergoing transimination reactions to achieve a nonequilibrium steady state (NESS) under light irradiation. Notably, the metastable *Z*-isomer is more susceptible to transimination, resulting in the formation of an imine in the *E*-isomeric state. In these systems, the imine photoswitches exhibit characteristics of an information ratchet, facilitating autonomous and directional cycling of the system under constant light irradiation. Using these properties, we demonstrated that energy can be stored in the metastable *Z*-isomers and in the system’s NESS constitution. We are currently exploring how such systems can be rationally perturbed further from equilibrium to amplify the difference between the equilibrium and NESS states. Additionally, we are investigating applications of these dynamic covalent ratcheting systems in the context of systems chemistry.^39^

## Data Availability

All data that support the findings of this study are included within the article and its Supporting Information and are also available from the authors upon reasonable request.

## Supporting Information Available

Additional experimental details; materials and methods; photoswitching properties; details of the kinetic studies; calculations of energy storage; control measurements; further analysis of the network; computational studies (PDF)
