# Supplementary material for: Photoswitchable Imines Drive Dynamic Covalent Systems to Nonequilibrium Steady States
Source: J Am Chem Soc. 2024 Jul 18;146(30):20720–7. doi: 10.1021/jacs.4c03817 (PMC11295185; doi:10.1021/jacs.4c03817)
Supplement: Supplementary file 1 — ja4c03817_si_001.pdf [file ja4c03817_si_001.pdf]

Supporting Information:

Photoswitchable Imines Drive Dynamic-Covalent Systems to Non-Equilibrium Steady States

Jiarong Wu[1,2] and Jake L. Greenfield[1,2]*

[1] Institut für Organische Chemie, Universität Würzburg, 97074 Würzburg, Germany

[2] Center for Nanosystems Chemistry (CNC), Universität Würzburg, 97074 Würzburg, Germany

Email: Jake.Greenfield@uni-wuerzburg.de

Table of Contents

1. Materials and Methods 3
  1.2 UV/vis and Photoswitching Studies 3
    1.2.1 Diode Array Setup 3
2. Synthesis and Characterisation 5
3. Photoswitching Properties 7
  3.1 Extinction Coefficient 7
  3.2 Thermal Half-life ($t_{1/2}$) 9
  3.3 Maximum Achievable %*Z* at Various Wavelengths 11
  3.4 Photoswitching Efficiency 12
4. Kinetic Studies 15
  4.1 Imine **1** + amine **C** without irradiation 17
  4.2 Imine **1** + amine **C** with 405 nm irradiation 19
  4.3 Imine **2** + amine **A** 23
5. Further Analysis of the System 24
  5.1 Cycle 1: Homo-aminal (**Aminal-1**) involved isomerization between *E*-**1** and *Z*-**1** 24
    5.1.1 From *E*-**1** to **Aminal-1** 25
    5.1.2 From *Z*-**1** to *E*-**1**, via **Aminal-1** 26
  5.2 Cycle 2: **Aminal-Mixed** involved isomerization and transimination from *Z*-**1** 30
  5.3 Cycle 3: Homo-aminal (**Aminal-2**) involved isomerization between *E*-**2** and *Z*-**2** 35
    5.3.1 From *E*-**2** to **Aminal-2** 36
    5.3.2 From *Z*-**2** to **Aminal-2**, to *E*-**2** 37
  5.4 Cycle 4: Mixed-aminal involved isomerization and transimination from *Z*-**2** 40
  5.5 Description of the Full System 41
  5.6 Flux of the Cycles 43
6. Energy Storage 45
7. Computational Studies 46
References 47

1. Materials and Methods

All reagents and solvents were purchased from commercial suppliers and used without further purification, unless specified.

UV/Vis Absorption. Spectra were recorded on a Jasco V-770 spectrophotometer, equipped with a Peltier cooling system (PAC-743R), or a diode array setup as detailed below in Section 1.2.1. Standard 10 mm and 1 mm pathlength quartz glass cuvettes (Fluorescence Cuvettes) from Hellma were used and a stirring bead was added to the 10 mm cuvettes. Measurements were run at 293.15 K, unless otherwise stated. Solutions for the UV/vis measurements were made using spectroscopy grade dry degassed solvents. A background measurement containing only the solvent was recorded before measuring samples. This background was subtracted from the sample data using Origin Software.

High-Resolution Mass Spectrometry (HR-MS). Electrospray ionisation (ESI) HR-MS spectrograms were recorded on a Bruker Daltonics microTOF focus instrument with a resolution of 18.000 FWHM. Samples were prepared in MeCN and the instrument was run in positive mode.

NMR Spectroscopy. NMR spectra were recorded at 298 K using a Bruker Avance HD III 400 MHz spectrometer automatically tuned and matched to the correct operating frequencies. The ^{13}C NMR spectra are broad-band proton decoupled. TopSpin 4.2 and MestReNova 14.3.2 were used to apply phase and baseline corrections. ^{1}H and ^{13}C NMR spectra were referenced to the residual solvent peak. Signals are reported in terms of chemical shift (ppm) and coupling constants (Hz). Abbreviations for multiplicity are as follows: s, singlet; d, doublet; t, triplet; m, multiplet.

1.2 UV/vis and Photoswitching Studies

The UV/vis absorption spectrum of the photoswitches reported in this paper were collected using either a JASCO V-770 spectrophotometer with a PAC-743R Peltier temperature controller or a home-built diode array setup (schematically shown below in Figure S1).

1.2.1 Diode Array Setup

The diode array setup schematically shown in Figure S1 was employed for Photostationary State (PSS), thermal half-life ($t_{1/2}$) and quantum yield measurements. The setup consists of an Ocean Insight DH-2000-FHS-DUV-TTL light source (190-2500 nm), connected to a Quantum Northwest QPod sample holder by 25 cm long premium fibre optics (Ocean Insight QP400-

025-SR-BX). The temperature of the QPod sample holder was controlled using a Quantum Northwest TC 125 temperature controller, which also controlled the stirring. An Ocean Insight Flame-S-XR1-ES diode array spectrometer was used to measure the absorbance spectrum of the sample. Pin-hole slits were employed as shown in the schematic along with an optical filter at the light source to reduce the probe light intensity (Thermo Oriel 50550).

LEDs of various wavelengths (see Table S1 below) were employed to induce photoisomerization. These LEDs were fitted with adjustable collimation adapters supplied by ThorLabs (either a SM1U with an LA4052-UV Fused Silica Plano Convex Lens with an anti-reflective coating 245-400 nm or a SM1U25-A with an anti-reflective coating 350-700 nm, depending on the wavelength of the LED used). The LEDs were operated at a constant current mode controlled by a ThorLabs DC2200 LED driver.

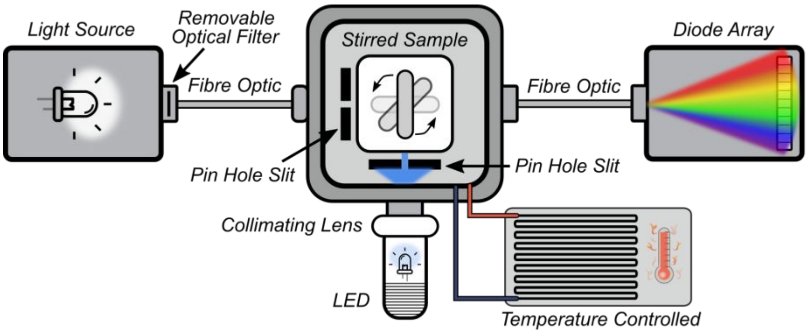

Figure S1. Schematic representation of the diode array setup up to measure the UV/vis absorption spectra and to perform photoswitching studies.

Table S1. Table displaying the models of ThorLabs mounted LEDs used in this study along with the nominal emission wavelength, the wavelength in which emission appears brightest to the human eye, and the bandwidth (full width at half maximum, FWHM).

LED model number (ThorLabs)	Wavelength (nm)	Bandwidth/FWHM (nm)
M340L5	340	10
M365L3	365	9
M385L3	385	11
M405L4	405	13
M430L5	430	17
M470L5	470	28

2. Synthesis and Characterisation

The precursor 2-(pyrrolidin-1-yl)benzaldehyde (**S1**) and imine **1** were synthesized based on previously published work from our group.[1]

Synthesis of *(E)-N-phenyl-1-(2-(pyrrolidin-1-yl)phenyl)methanimine*, 2

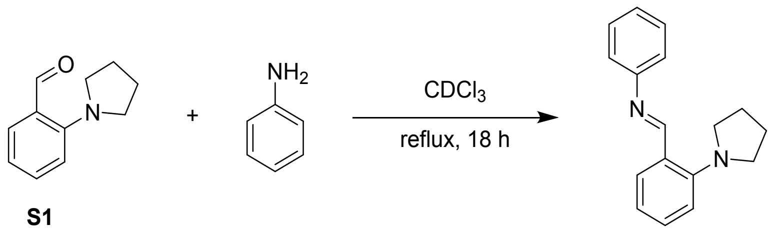

Figure S2. The formation of imine **2** from **S1** and aniline.

In oven-dried glassware, a solution of aniline (139.7 mg, 1.5 mmol, 3 eq) and **S1** (125.2 mg, 0.5 mmol, 1.0 eq) in $CDCl_3$ (2.0 mL) was prepared under N_2. The mixture was heated to reflux for 18 hours. The reaction progress was monitored using ^1H NMR spectroscopy, and completion was determined by the complete consumption of the aldehyde. The solvent and excess aniline were then removed under high vacuum. The resulting orange oil was obtained in quantitative yield without further purification. ^1H NMR (400 MHz, 298 K, MeCN-d_3): δ 8.77 (s, 1H), 7.91 (ddt, J = 7.8, 1.8, 0.4 Hz, 1H), 7.45 – 7.36 (m, 2H), 7.33 (ddd, J = 8.4, 7.1, 1.8 Hz, 1H), 7.25 – 7.16 (m, 3H), 6.96 (dd, J = 8.4, 1.1 Hz, 1H), 6.91 – 6.84 (m, 1H), 3.43 – 3.23 (m, 4H) ppm; ^{13}C NMR (101 MHz, 298 K, MeCN-d_3): δ 160.60, 153.46, 151.42, 132.10, 129.76, 129.62, 125.94, 125.05, 121.39, 119.16, 115.96, 53.49, 25.84 ppm; HR ESI-MS (MeCN) for $[C_{17}H_{18}N_2+H]^+$: m/z calcd: 251.1543; found: 251.1543; 0.3 ppm error.

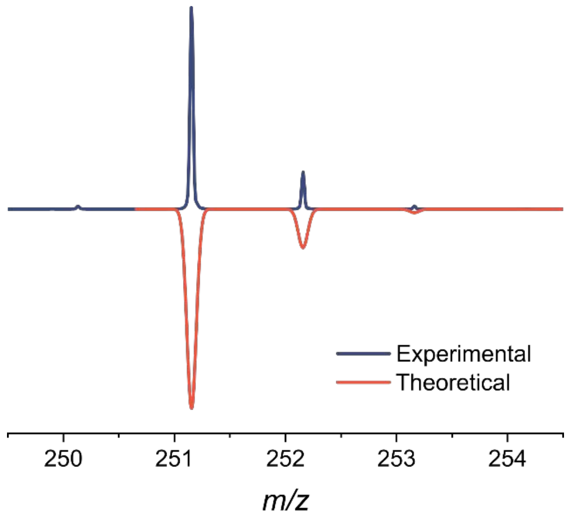

Figure S3. The HR-MS (ESI) of imine **2**. The theoretical isotope pattern is shown in red and reflected in the x-axis. The experimental and theoretical traces are normalized for comparison.

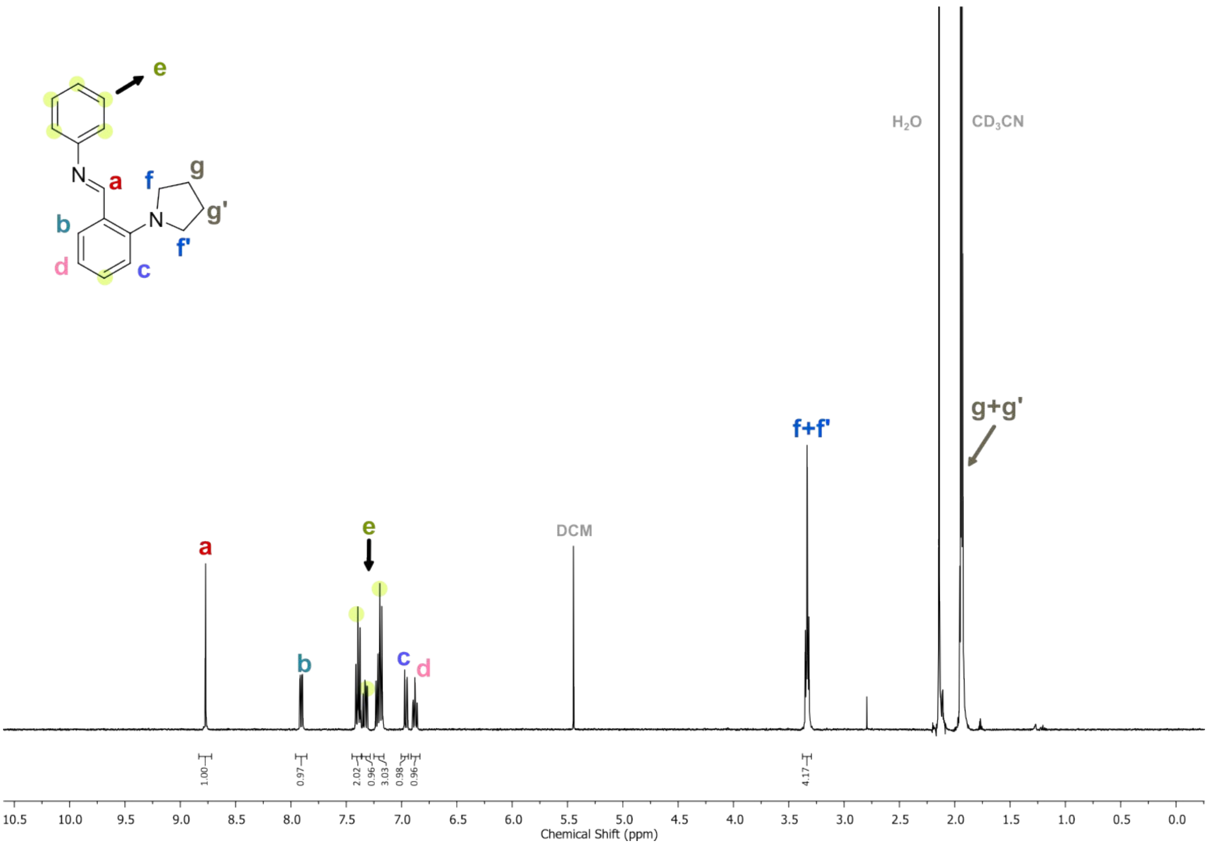

Figure S4. ^{1}H NMR (400 Hz, 298 K, MeCN-d_3) of imine *E*-**2**.

3. Photoswitching Properties

The photoswitching properties of imine **1** were previously investigated by our group.[1] The newly introduced imine in this work (imine **2**) was characterized using the same set-up and methods. The maximum absorption wavelengths of *E*-isomer (λ_{max}), extinction coefficients at λ_{max}, and the thermodynamic properties of these four imines are listed in Table S2.

Table S2. The wavelengths of maximum absorbance (λ_{max}), the extinction coefficients at λ_{max}, and the thermodynamic properties of imine **1** and imine **2**.

	**λ_{max}** (nm)	**Extinction Coefficient at λ_{max}** (M^{-1} cm^{-1})	**$t_{1/2}$ at 20 °C**	**$\Delta G^{‡}$ at 295 K** ($kJ\cdot mol^{-1}$)	**$\Delta H^{‡}$** ($kJ\cdot mol^{-1}$)	**$\Delta S^{‡}$** ($J\cdot K^{-1}\cdot mol^{-1}$)	**Ref.**
**1**	370	8310	22.1 min	90.18	86.27	−13.25	1
**2**	376	6830	2.7 s	75.56	63.72	−40.15	-

3.1 Extinction Coefficient

The UV/vis spectra in this work were measured using a Jasco V-770 UV/vis/NIR spectrophotometer at various concentrations. According to Beer-Lambert Law,[2] there exists a linear relationship between the absorbance and the concentration of the solution, which can be expressed as:

$$A = \epsilon l c$$

In this equation, "A" represents the absorbance in absorbance units (a.u.), "l" is the optical path length, i.e., the length of the cuvette used for the absorbance measurement (1 cm), and "c" is the molar concentration in moles per litre (M). From the absorption spectra (Figure S5), the molar extinction coefficient (ε) at the specific wavelength (λ_{max}) can be determined by fitting a linear calibration curve of the absorbance versus concentration.

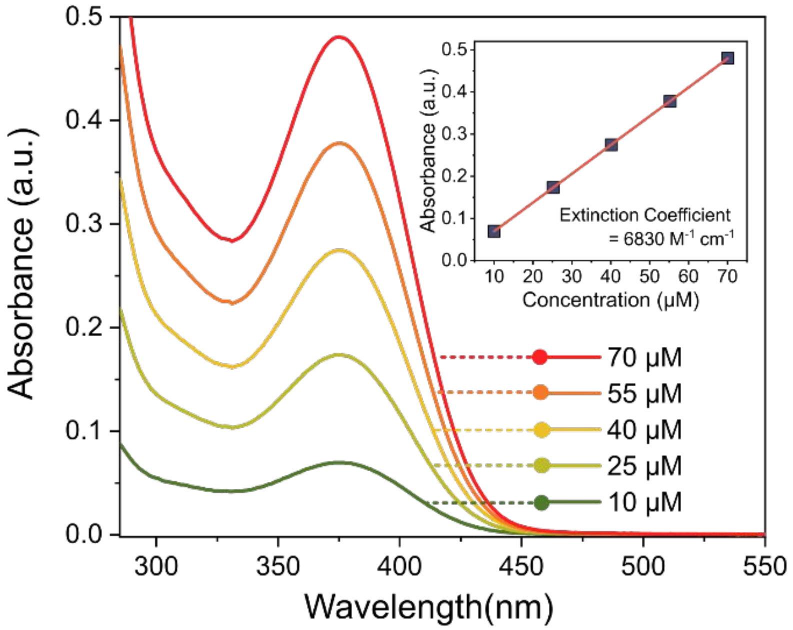

Figure S5. The UV/vis absorption spectra of imine *E*-**2** were measured at 20 °C in acetonitrile (l = 1 cm), at different concentrations. The calibration curve, shown in the top right corner of the figure, was plotted at the wavelength of λ_{max} (376 nm) to determine the slope, which represents the extinction coefficient at this wavelength.

3.2 Thermal Half-life ($t_{1/2}$)

The *Z*-to-*E* isomerism kinetics of imine **2** was investigated using the diode array setup (Section 1.2.1 of the Supporting Information). The measurements were conducted in the solution state using acetonitrile as the solvent, and a range of temperatures was employed to enable Eyring plots to be constructed. The isomerization of the *E*-isomer to the *Z*-rich system was induced by irradiation with 365 nm LED light. Assuming a first-order reaction, the rate constants (k) for the thermodynamic switching process from the *Z*-rich system to the *E*-isomer at different temperatures were determined by fitting the absorbance changes over time to an exponential (Figures S6).

The thermal half-life ($t_{1/2}$) of *Z*-isomer at 20 °C was calculated based on the rate constant:

$$t_{1/2} = \frac{ln2}{k}$$

Utilizing the linearized form of the Eyring equation presented below:[3]

$$ln\,\frac{k}{T} = \frac{-\Delta H^{\ddagger}}{R}\cdot\frac{1}{T} + ln\,\frac{k_B}{h} + \frac{\Delta S^{\ddagger}}{R}$$

The values for enthalpy of activation ($\Delta H^{\ddagger}$) and entropy of activation ($\Delta S^{\ddagger}$) in Table S2 can be determined from the kinetic data at different temperatures by linear fitting (Figure S6f). Gibbs energy of activation (listed in Table S2) can be calculated using the equation below:

$$\Delta G^{\ddagger} = \Delta H^{\ddagger} - T\Delta S^{\ddagger}$$

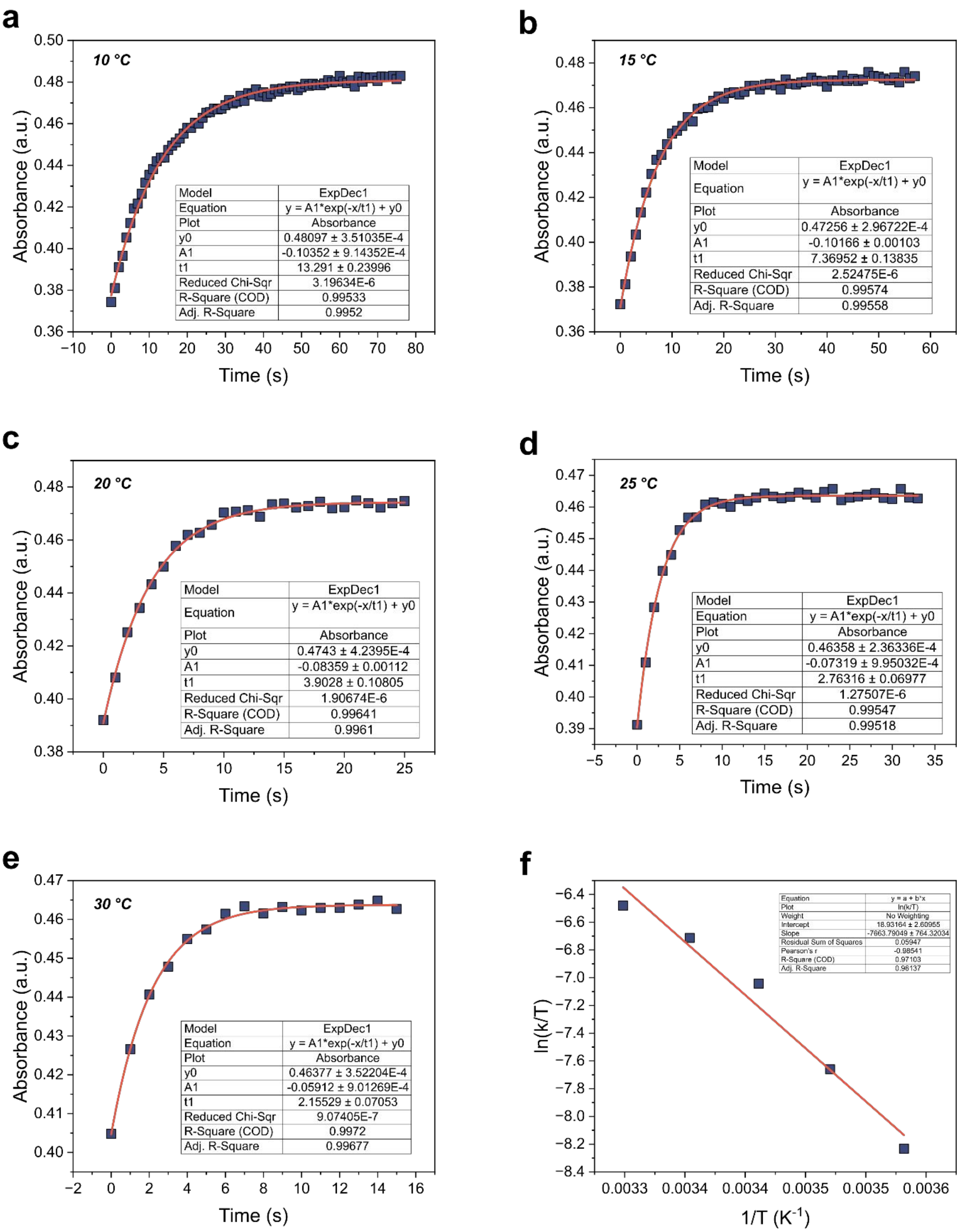

Figure S6. The change in absorbance of **2** at the λ_{max} of the *E*-isomer over time at **a**, 10 °C, **b**, 15 °C, **c**, 20 °C, **d**, 25 °C, **e**, 30 °C, with the increase in absorbance relating to the thermal isomerization of the *Z*-isomer to the *E*-isomer. The sample was irradiated with a 365 nm LED for 5 minutes in acetonitrile before the measurement started. The exponential fittings were applied to determine the rate constants and thus the thermal half-lives. **f,** The Eyring plot of **2** (70 µM in MeCN), was generated using the rate constants calculated at different temperatures. The fitted parameters were used to estimate the enthalpy of activation ($\Delta H^{‡}$), entropy of activation ($\Delta S^{‡}$), and Gibbs energy of activation ($\Delta G^{‡}$) listed in Table S2.

3.3 Maximum Achievable %*Z* at Various Wavelengths

The photostationary state (PSS) is a photodynamic equilibrium achieved between *E*- and *Z*-isomers after exposure to a specific wavelength of light. However, for imine **2**, the short $t_{1/2}$ (as shown in Section 3.2 of the Supporting Information) preclude the measurement of the %*Z* at the PSS due to a significant amount of *Z*-to-*E* thermal reversion taking place during photoisomerism. Thus, here we use the maximum achievable %*Z* isomer to determine the properties of imine **2** as the PSS is not reached under these conditions. It is important to note that our setup, shown schematically in Figure S1, facilitates the rapid acquisition of the UV/vis spectra: 50 ms integration time, average of 10 spectra, resulting in a spectrum being recorded every 0.5 s. The combination of the high intensity of irradiation, rapid spectral acquisition, and temperature control limits the amount of time between irradiation and spectral acquisition, providing a relatively more reliable measurement of the %*Z* isomer at the given irradiation wavelength, intensity, and temperature.

The irradiation was conducted using LED lights ranging from 340 nm to 470 nm, as detailed in Table S1. To determine the %*Z* isomer, Fischer's approach was employed.[4] This method analyses the absorption spectra of the pure *E*-isomer (before irradiation) and the mixture after irradiation at two specific wavelengths. From this, the UV/vis of the pure *Z*-isomer was predicted (see below). Finally, linear interpolation between the dark (pure *E*) and predicted *Z*-isomer spectra enables the determination of the %*Z* isomer at other irradiation wavelengths.

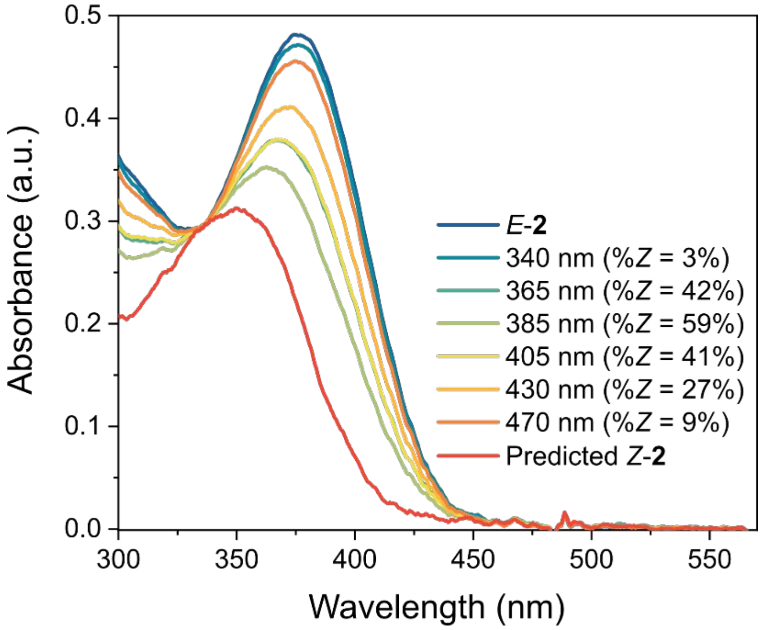

Figure S7. The UV/vis spectra of **2** (70 µM in MeCN) under different irradiation conditions at 20 °C, alongside the predicted *Z*-isomer spectrum. Note that the LEDs here were operated using the maximum power output, different to that used for generating the NESS and that reported in Section 3.4. Note that for 405 nm here, 900 mA at 90% power was used. These samples were constantly stirred under irradiation.

3.4 Photoswitching Efficiency

The irradiation setup employed in this study was identical to that described in our previous work.[1] The photon fluxes provided by the light source with the wavelengths of 340 nm, 365 nm, 385 nm, 405 nm, and 430 nm are listed in Table S3.

Table S3. The photon fluxes provided by light source with different wavelengths respectively.[1]

**Wavelength**	**Photon Flux** (Photons/s)
340 nm	4.25×10^{14}
365 nm	6.32×10^{14}
385 nm	7.38×10^{14}
405 nm	6.29×10^{14}
430 nm	7.61×10^{14}

The sample of imine **2** was prepared in acetonitrile at room temperature. The irradiation and measurements were performed in the diode array setup simultaneously, with the same irradiation conditions as the calibration process[5,6] as detailed in our previous work:[1] 340 nm (500 mA, 90%), 365 nm (200 mA, 20%), 385 nm (200 mA, 20%), 405 nm (200 mA, 20%), 430 nm (200 mA, 20%), respectively. However, the short thermal half-life of the *Z*-isomer in imine **2** limits the effectiveness of this approach. Under these conditions, the photon flux is insufficient to overcome the simultaneous thermal relaxation of the *Z*-isomer back to the *E*-isomer. Consequently, reliable measurement of quantum yields (which quantify the efficiency of photoisomerization) becomes challenging.

Alternatively, to assess the photoswitching efficiency of imines **1** and **2**, and also to evaluate the influence of amine concentration under conditions identical to those used in the kinetic studies described in Section 4.2 of the Supporting Information, control experiments were conducted as shown in Figure S9. The samples of (a) imine **1** (6 mM) alone, (b) imine **1** (6 mM) with amine **A** (6 mM), (c) imine **1** (3 mM) alone, (d) imine **1** (3 mM) with amine **A** (3 mM), (e) imine **2** (2.5 mM) alone, and (f) imine **2** (2.5 mM) with amine **C** (0.3 M) were prepared in MeCN at room temperature. A 1 mm cuvette was used for the UV/vis measurements due to the higher concentrations used and the measurements were performed using the diode array setup (Section 1.2.1, Supporting Information). The chosen wavelength (405 nm) of irradiation and the power output (500 mA, 90%) are also consistent with the irradiation conditions used in the kinetic experiments. For imine **1**, the PSSs were achieved within 30 seconds, indicating efficient photoisomerization. However, for imine **2**, due to its fast thermal relaxation from *Z*- to *E*-state at room temperature, only around 30% of **2** exists as the *Z*-isomer under these

conditions. The addition of amine has minimal impact on the achievable *Z*-isomers for both imine **1** and **2**. Notably, the maximum achieved %*Z*-isomers in Section 3.3 of imine **2** were measured using the maximum power output of each LED (Table S1).

Note that in these measurements, higher concentrations are used, thus limiting the available spectral range that can be analysed reliably. We therefore limit our analysis to the wavelength regions that afford an absorbance ≤1. Moreover, given this limited spectral range and its distance from the λ_{max} of the *E*/*Z* isomers, larger deviations in the estimated PSS are to be expected. Thus, as no substantial change (± 10%) in the amount of *Z*-isomer was observed compared to measurement at 150 µM concentrations (32% of **2** as *Z*-isomer as shown in Figure S8, compared to 30% of **2** as *Z*-isomer as shown in Figure S9e), we assume that the amount of *Z*-isomer is unchanged and use this value in our quantitative analysis. It is also worth highlighting that the useful property of negative photochromism exhibited by these imine-based photoswitches[1] enables efficient photoswitching even at such high concentrations.

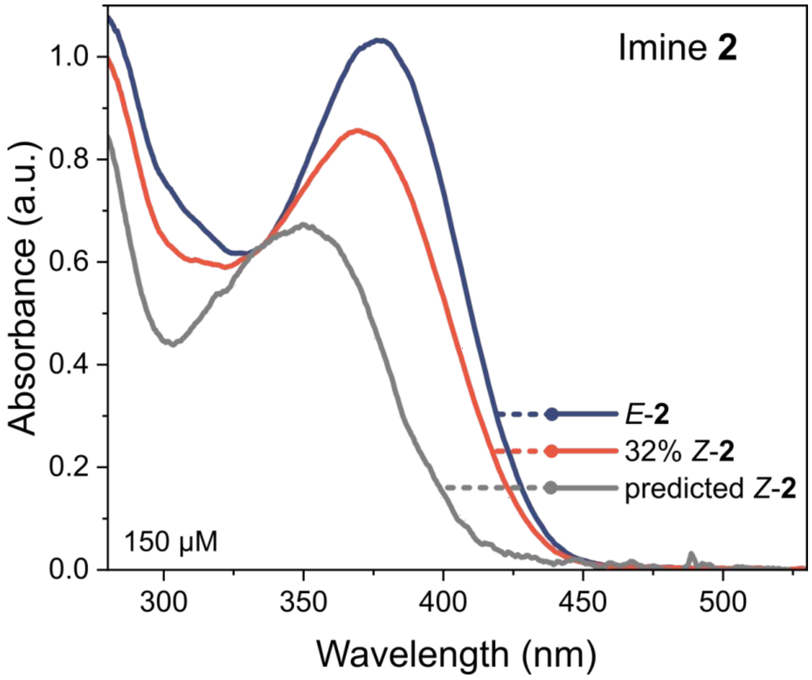

Figure S8. The UV/vis spectra of imine **2** (150 µM in MeCN) at room temperature. The blue curve was recorded before irradiation, the red curve was recorded after irradiation for 5 minutes, and the grey curve is normalized predicted spectrum of *Z*-isomer. The power condition used here is 500 mA, 90%, the same as the NESS measurement in Section 4.2, Supporting Information. Note that stirring was not used in this measurement due to not being able to achieve a PSS; mixing of the sample resulted in a lower amount of *Z*-isomer due to the constant exchange of *E*-**2** outside of the irradiation zone with *Z*-**2** in the irradiation zone. The NMR measurements were also not stirred.

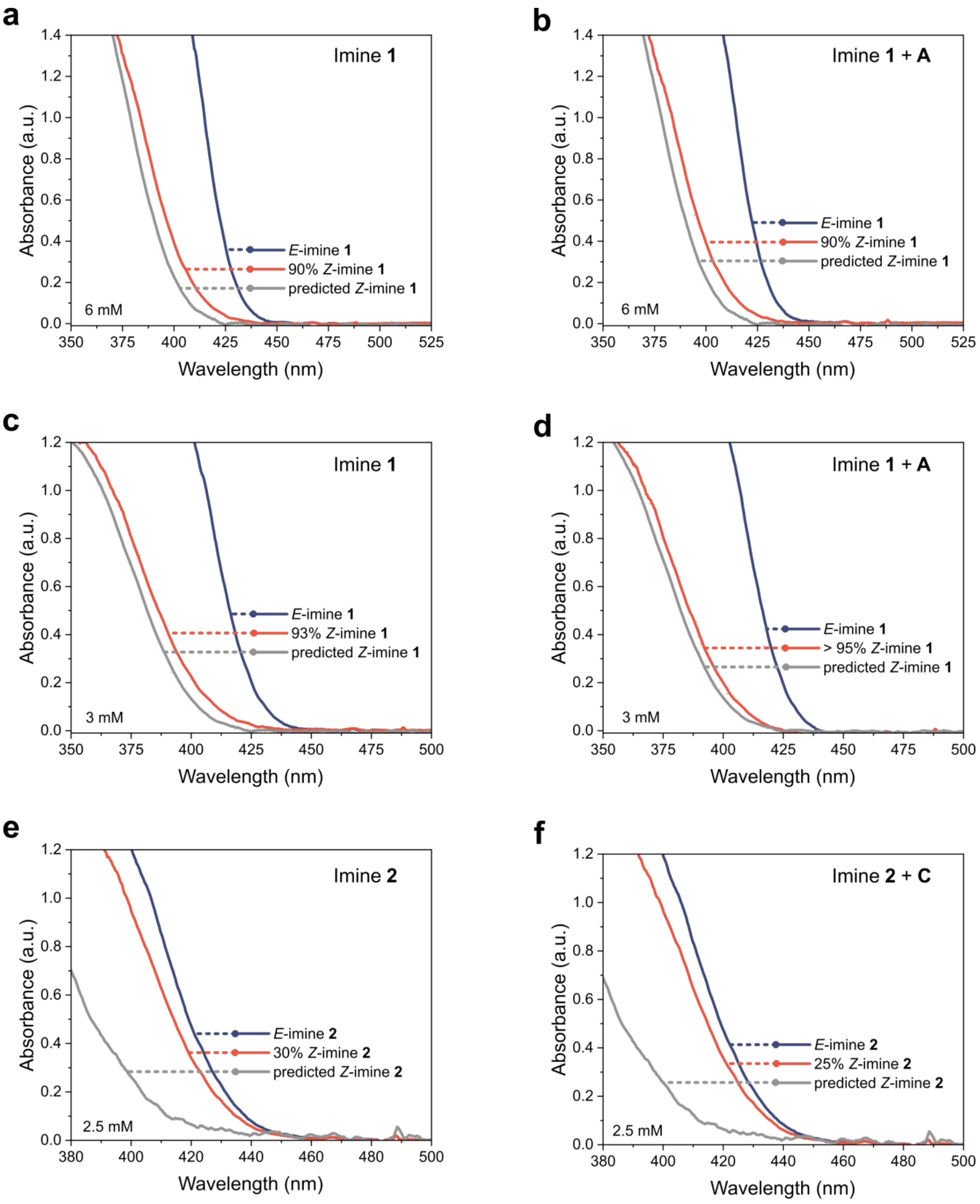

Figure S9. The UV/vis spectra of **a**, imine **1** (6 mM); **b**, imine **1** (6 mM) + amine **A** (6 mM); **c**, imine **1** (3 mM); **d**, imine **1** (3 mM) + amine **A** (3 mM); **e**, imine **2** (2.5 mM); **f**, imine **2** (2.5 mM) + amine **C** (0.3 M), in MeCN at room temperature. The blue curves were recorded before irradiation, the red curves were recorded after irradiation for 5 minutes of each sample, and the grey curves are normalized predicted spectra of *Z*-isomers. The power condition used here is 500 mA, 90%, the same as the NESS measurement in Section 4.2, Supporting Information.

4. Kinetic Studies

To understand the dynamic processes, kinetic studies were conducted to investigate the transimination reactions. The determined rate constants and equilibrium constants are listed in Table S4. The calculated equilibrium constants were determined by the composite rate constants from the fit of the kinetic data (k_f / k_b, see below), and the observed equilibrium constants were determined from the integration of ^{1}H NMR signals for the equilibrated samples. The good agreement between the calculated and observed constants suggests a reliable fit of the kinetic data. Notably, since the reaction under irradiation reaches a NESS instead of true thermodynamic equilibrium, we use “apparent constants” here to describe their properties.

Table S4. Overview of the rate constants and equilibrium constants investigated.

**1 + Aniline (C)**	**k_f (M^{-1} s^{-1})**	**k_b (M^{-1} s^{-1})**	**K (k_f / k_b)**	**K**	**R^2**
**Without irradiation**	3.2×10^{-5}	7.7×10^{-3}	4.19×10^{-3}	3.86×10^{-3}	0.9822
**With 405 nm irradiation**	3.6×10^{-4} *	5.3×10^{-2} *	6.75×10^{-3} *	6.62×10^{-3} *	0.9928

*The constants under irradiation are described as apparent constants.

In general, the transimination reaction taking place in the system can be expressed as:

$$AB + B' \leftrightarrow AB' + B$$

where AB and AB’ are imines, and B and B’ are amines. The composite rate constant for the forward reaction is denoted as k_f, while k_b represents the composite rate constant for the backward reaction.

In this kind of reversible reaction, the observed rate is the sum of the forward and backward reaction connected by the equilibrium constant (K):

$$\frac{d[AB']}{dt} = k_f \cdot ([AB]_0 - [AB']) \cdot ([B']_0 - [AB']) - k_b \cdot [AB']^2$$

$$K = \frac{k_f}{k_b}$$

Note that here, [B] is equal to [AB’], thus the $[AB']^2$ term, as our starting conditions in our experiments initially only include species AB and B’.

When the initial concentration of AB equals to a ($[AB]_0 = a$), and n equivalents of B' is added ($[B']_0 = na$), at time t, the concentration of product AB' is x ($[AB'] = x$):

	AB	$+$	B'	$\leftrightarrow$	AB'	$+$	B
$t = 0$	a		na		0		0
$t = t$	$a - x$		$na - x$		x		x

$$\frac{dx}{dt} = k_f(a - x)(na - x) - k_b x^2$$

$$\frac{dx}{dt} = \left(k_f - k_b\right)x^2 - a(n + 1)k_f x + k_f na^2$$

$$\int_0^x \frac{dx}{\left(k_f - k_b\right)x^2 - a(n + 1)k_f x + k_f na^2} = \int_0^t dt$$

In the equation, a, n, x, and t can be determined by experimental data, the composite rate constants (k_f and k_b) can be calculated from the fitting (Figure S12, and S15), using Fit ordinary differential equation (Fit ODE.opx) in OriginLab.

4.1 Imine 1 + amine C without irradiation

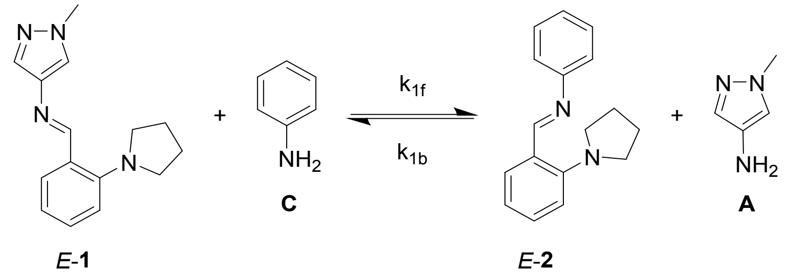

Figure S10. Overview of the transimination reaction between *E*-**1** and **C** to yield *E*-**2** and **A** in the absence of light irradiation.

A solution of imine **1** (6 mM in CD_3CN) was prepared in the dark to avoid photoisomerization under ambient light. Then, 50 equivalents of aniline were added, marking the reaction's start time (t_0 = 0 min). The reaction progress was monitored using ^{1}H NMR spectroscopy over time (Figure S11). The observed concentrations of imine **1** and **2** in Figure S11 were calculated by integrating their normalized ^{1}H NMR signals and multiplying by the initial concentration of imine **1**. The composite rate constants were obtained from the fitting. The equilibrium constant K_{eq} was obtained from the integration of ^{1}H NMR signals of the equilibrated sample.

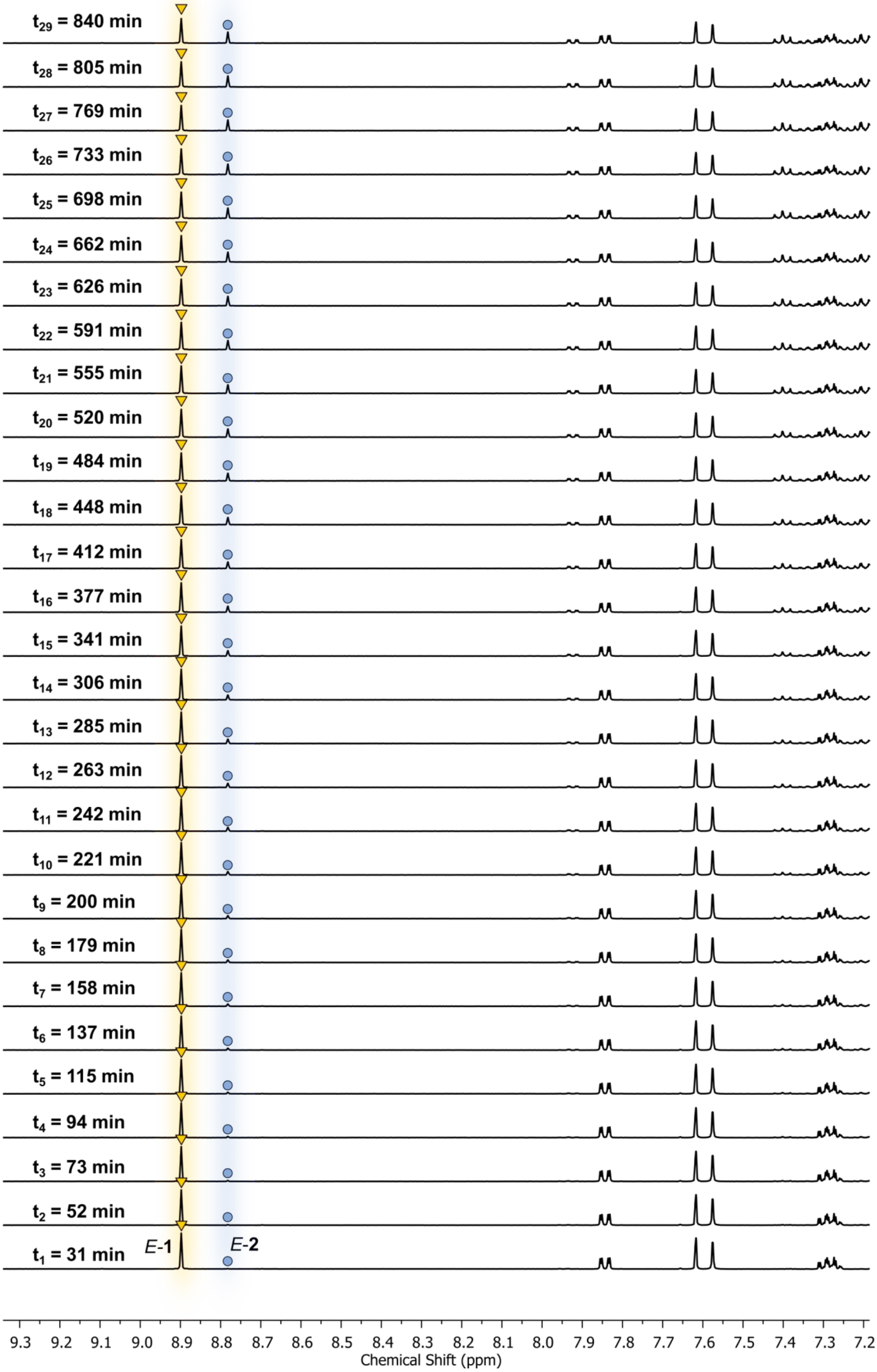

Figure S11. The ¹H NMR (400 Hz, 298 K, MeCN-d_3) of imine **1** (6 mM), following the addition of 50 equivalents of aniline (**C**). The NMR sample was kept in the dark during this study.

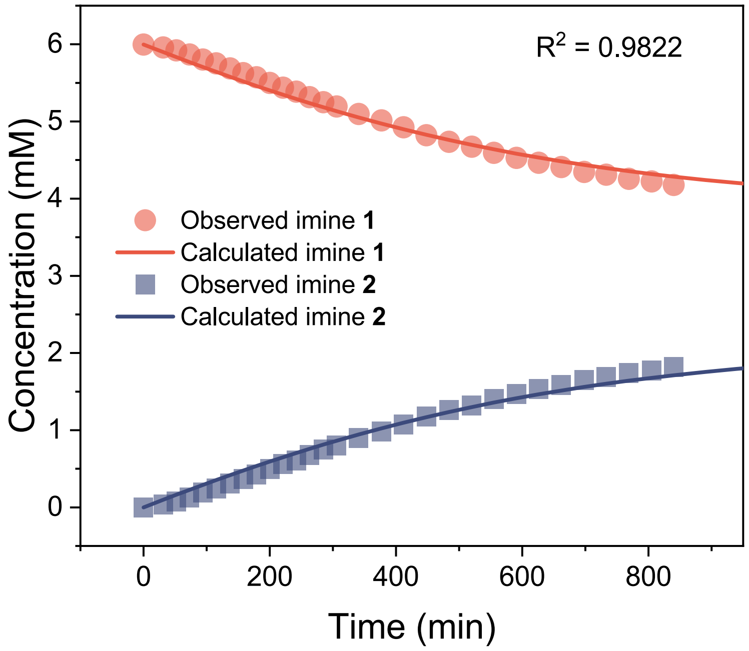

Figure S12. Imine **1** + 50 equivalents of aniline (**C**), without irradiation. The observed concentrations were determined by ^{1}H NMR (the initial concentration of imine **1** was 6 mM in CD_3CN). From the fitting, the composite rate constants: $k_{1f} = 3.2 \times 10^{-5}\, M^{-1}\, s^{-1}$, $k_{1b} = 7.7 \times 10^{-3}\, M^{-1}\, s^{-1}$, the calculated equilibrium constant $K_{eq}\,(\frac{k_{1f}}{k_{1b}}) = 4.2 \times 10^{-3}$, compared to the observed equilibrium constant $K_{eq} = 3.89 \times 10^{-3}$.

4.2 Imine 1 + amine C with 405 nm irradiation

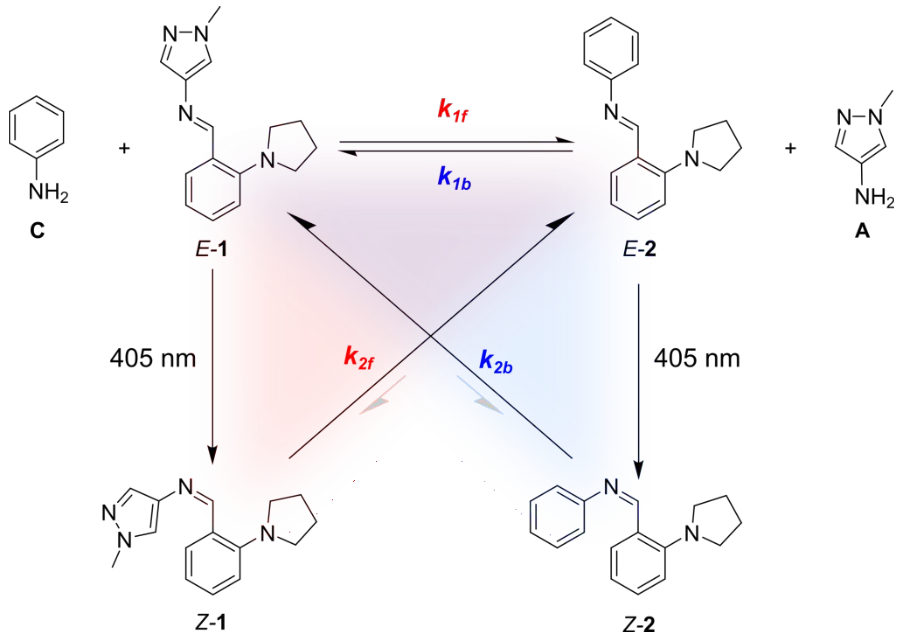

Figure S13. Schematic representation of the dynamic-covalent system comprising of *E*-**1**, *Z*-**1**, *E*-**2**, *Z*-**2**, **C** and **A**. The color shading indicates closed, interdependent cycles present in the system.

To investigate the effect of light irradiation on the reaction between imine **1** and aniline (**C**), as well as the reaction between imine **2** and **A** (as the *Z*-isomers may exhibit higher reactivity towards amines compared to the *E*-isomer), kinetic studies were conducted under 405 nm light. In this experiment, a solution of imine **1** (6 mM in CD_3CN) was prepared in dark to avoid photoisomerization under ambient light. The sample was irradiated with 405 nm light and 50 equivalents of aniline (**C**) were immediately added, marking the reaction's start time (t_0 = 0 min). The reaction progress was monitored using ^{1}H NMR spectroscopy over time (Figure S14). Notably, the time intervals between measurements consisted of a 10-minute irradiation period followed by 11 minutes for NMR acquisition. The relatively low quantum yields of these imine photoswitches precluded the use of in-situ NMR irradiation measurements.[1] The observed concentrations of imine **1** and **2** in Figure S15 were calculated by integrating their normalized ^{1}H NMR signals and multiplying by the initial concentration of imine **1**. The apparent composite rate constants were calculated from the fitting. The observed apparent equilibrium constant (K_{ap}) was obtained from integrating ^{1}H NMR signals of NESS sample. Note that signals assigned to a population of *Z*-**1** were observed in the ^{1}H NMR spectra attributed to the relatively long-lived *Z*-isomer ($t_{1/2}$ of 22.1 min at 20 °C).[1]

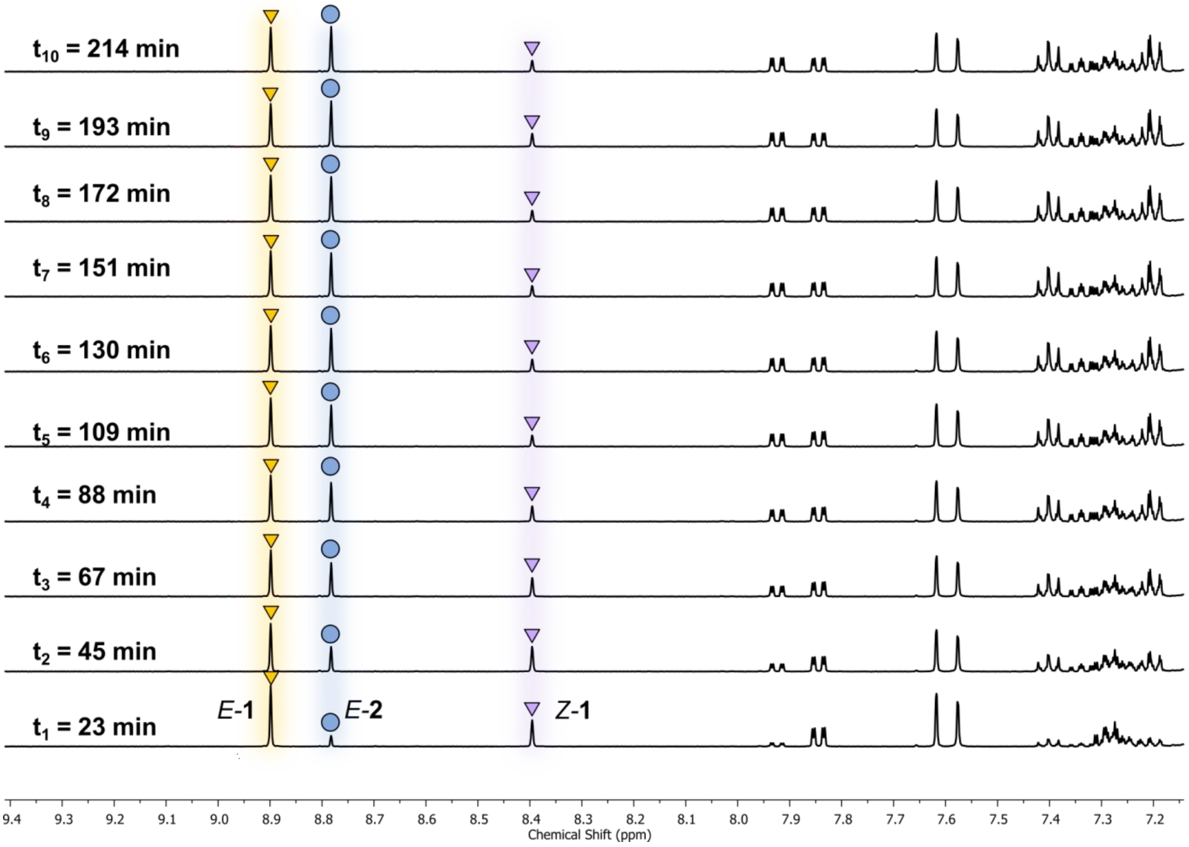

Figure S14. The ^{1}H NMR (400 Hz, 298 K, MeCN-d_3) of imine **1** (6 mM), following the addition of 50 equivalents of aniline (**C**). Each spectrum was acquired following 10 minutes of irradiation with 405 nm light.

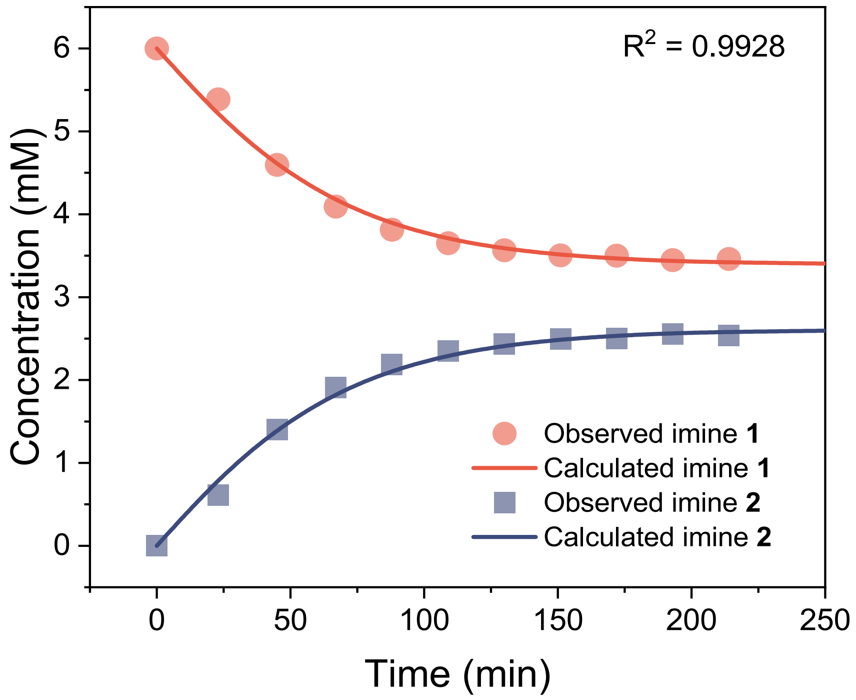

Figure S15. Imine **1** + 50 equivalents of aniline (**C**), with 405 nm irradiation. The observed concentrations were determined by ^{1}H NMR (the initial concentration of imine **1** was 6 mM in CD_3CN). From the calculation, the apparent composite rate constants: $k_{hv,f} = 3.6 \times 10^{-4}\ M^{-1}\ s^{-1}$, $k_{hv,b} = 5.3 \times 10^{-2}\ M^{-1}\ s^{-1}$, the calculated apparent equilibrium constant K_{ap} $(\frac{k_{hv,f}}{k_{hv,b}}) = 6.75 \times 10^{-3}$, compared to the observed apparent equilibrium constant $K_{ap} = 6.62 \times 10^{-3}$.

Due to the presence of both *E*- and *Z*-isomers in the system (*E*-**1**, *Z*-**1**, *E*-**2**, *Z*-**2**) under 405 nm irradiation, the reaction from imine **1** to imine **2** involves multiple pathways. For the forward reaction (from imine **1** to imine **2**), the rate consists of two contributions: from *E*-**1** to *E*-**2** and from *Z*-**1** to *E*-**2**:

$$r_{hv,f} = r_{1f} + r_{2f}$$

where $r_{hv,f}$ is the rate for the reaction from imine **1** to imine **2** under irradiation, r_{1f} is the rate of the reaction from *E*-**1** to *E*-**2**, and r_{2f} is the rate of the reaction from *Z*-**1** to *E*-**2**.

$$k_{hv,f} \cdot [\mathbf{1}] \cdot [\boldsymbol{C}] = (k_{1f} \cdot [E - \mathbf{1}] \cdot [\boldsymbol{C}]) + (k_{2f} \cdot [Z - \mathbf{1}] \cdot [\boldsymbol{C}])$$

$$k_{hv,f} \cdot [\mathbf{1}] = (k_{1f} \cdot [E - \mathbf{1}]) + (k_{2f} \cdot [Z - \mathbf{1}])$$

where $k_{hv,f}$ is the apparent composite rate constant for the reaction from imine **1** to imine **2** under irradiation, calculated from the measurements, k_{1f} is the composite rate constant of the reaction from *E*-**1** to *E*-**2**, and k_{2f} is the rate constant of the reaction from *Z*-**1** to *E*-**2**.

The irradiation condition used in this experiment leads to a photostationary state of imine **1**, containing 95% of **1** as the *Z*-isomer (Section 3.4, Supporting Information):

$$k_{hv,f} = (0.05 \cdot k_{1f}) + (0.95 \cdot k_{2f})$$

Given that $k_{1f} = 3.2 \times 10^{-5}\ M^{-1}\ s^{-1}$ (as shown in Figure S12), and $k_{hv,f} = 3.6 \times 10^{-4}\ M^{-1}\ s^{-1}$ (as shown in Figure S15):

$$k_{2f} = 3.8 \times 10^{-4}\ M^{-1}\ s^{-1}$$

Similarly, the rate for the backward reaction (from imine **2** to imine **1**) also consists of two parts: *E*-**2** to *E*-**1** and *Z*-**2** to *E*-**1**:

$$r_{hv,b} = r_{1b} + r_{2b}$$

where $r_{hv,b}$ is the rate for the reaction from imine **2** to imine **1** under irradiation, r_{1b} is the rate of the reaction from *E*-**2** to *E*-**1**, and r_{2b} is the rate of the reaction from *Z*-**2** to *E*-**1**.

$$k_{hv,b} \cdot [\mathbf{2}] \cdot [\boldsymbol{A}] = (k_{1b} \cdot [E - \mathbf{2}] \cdot [\boldsymbol{A}]) + (k_{2b} \cdot [Z - \mathbf{2}] \cdot [\boldsymbol{A}])$$

$$k_{hv,b} \cdot [\mathbf{2}] = (k_{1b} \cdot [E - \mathbf{2}]) + (k_{2b} \cdot [Z - \mathbf{2}])$$

where $k_{hv,b}$ is the apparent composite rate constant for the reaction from imine **2** to imine **1** under irradiation, calculated from the measurements, k_{1b} is the composite rate constant of the reaction from *E*-**2** to *E*-**1**, and k_{2b} is the rate constant of the reaction from *Z*-**2** to *E*-**1**.

The irradiation condition used in this experiment leads to a NESS of imine **2**, containing 30% of **2** as the *Z*-isomer (Section 3.4, Supporting Information):

$$k_{hv,b} = (0.7 k_{1b}) + (0.3 k_{2b})$$

Given that $k_{1b} = 7.7 \times 10^{-3}\ M^{-1}\ s^{-1}$ (as shown in Figure S12), and $k_{hv,b} = 5.3 \times 10^{-2}\ M^{-1}\ s^{-1}$ (as shown in Figure S15):

$$k_{2b} = 1.6 \times 10^{-1} M^{-1}\ s^{-1}$$

4.3 Imine 2 + amine A

To confirm the reversibility of the reaction and validate the equilibrium constant determined in Section 4.1 (Supporting Information), the experiments of the reverse reaction as shown in Figure S10 were conducted. The first experiment investigated the direct reaction with amine **A**, by preparing the solution of imine **2** (6 mM in CD_3CN) with 1 equivalent of amine **A**. The reaction was allowed to reach equilibrium, achieving a value for the equilibrium constant of 3.87×10^{-3}. However, due to the low concentration of remaining imine **2** at equilibrium (~2.5%), errors from integration of the ^{1}H NMR signals become not negligible in this case.

To address this limitation, a second experiment was conducted using a mixture containing imine **2** (6 mM), 50 equivalents of amine **C** (0.3 M), and 1 equivalent of amine **A** (6 mM) in CD_3CN. This second experiment also reached equilibrium with an equilibrium constant of 3.92×10^{-3}, which is in close agreement with the value previously observed ($K_{eq} = 3.89 \times 10^{-3}$ as shown in Figure S12).

5. Further Analysis of the System

Since the system displays a degree of autocatalytic behaviour (see brief discussion in main manuscript), the specific pathway for this autocatalysis is still unknown. To gain a preliminary understanding despite these limitations, a simplified model is applied here. In this model, the system is divided into four cycles, considering the potential involvement of the aminal intermediates in the reaction network.

5.1 Cycle 1: Homo-aminal (**Aminal-1**) involved isomerization between *E*-**1** and *Z*-**1**

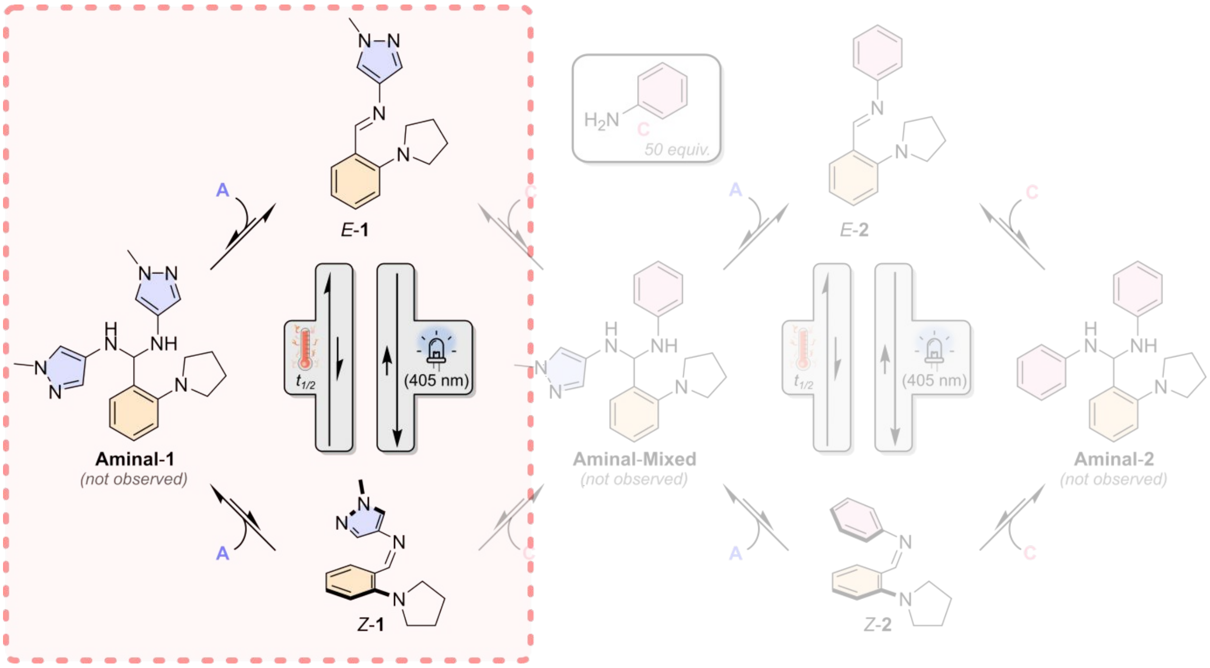

Figure S16. Schematic representation of the full system, highlighting Cycle 1. Grey shaded boxes indicate unimolecular reaction pathways.

The photoisomerization from *E*-**1** to *Z*-**1** and the thermal relaxation back from *Z*-**1** to *E*-**1** are both unimolecular processes.[1] However, the presence of excess amine in the system could potentially lead to biasing aminal formation, which could influence the rate or equilibrium of the isomerization in both directions. To investigate the potential impact of **Aminal-1** formation, we performed control experiments by adding aminopyrazole (**A**) in varying degrees of excess into the solution of imine **1**.

5.1.1 From *E*-1 to Aminal-1

As shown in Figure S17, varying amounts of amine **A** was added to the solution of imine **1** at molar ratios of (a) 1:1, (b) 1:10, and (c) 1:50. ^{1}H NMR analysis after 1 and 24 hours did not reveal any signal corresponding **Aminal-1**. This suggests both the slow formation and rapid reaction of the aminal intermediate, leaving the equilibrium heavily on the side of the imines. Additionally, the absence of the *Z*-**1** isomer, even with a 50-fold excess of amine **A**, indicates a strong thermodynamic preference for *E*-**1**. We infer that the *E*-**1** to *Z*-**1** in the absence of light is negligible.

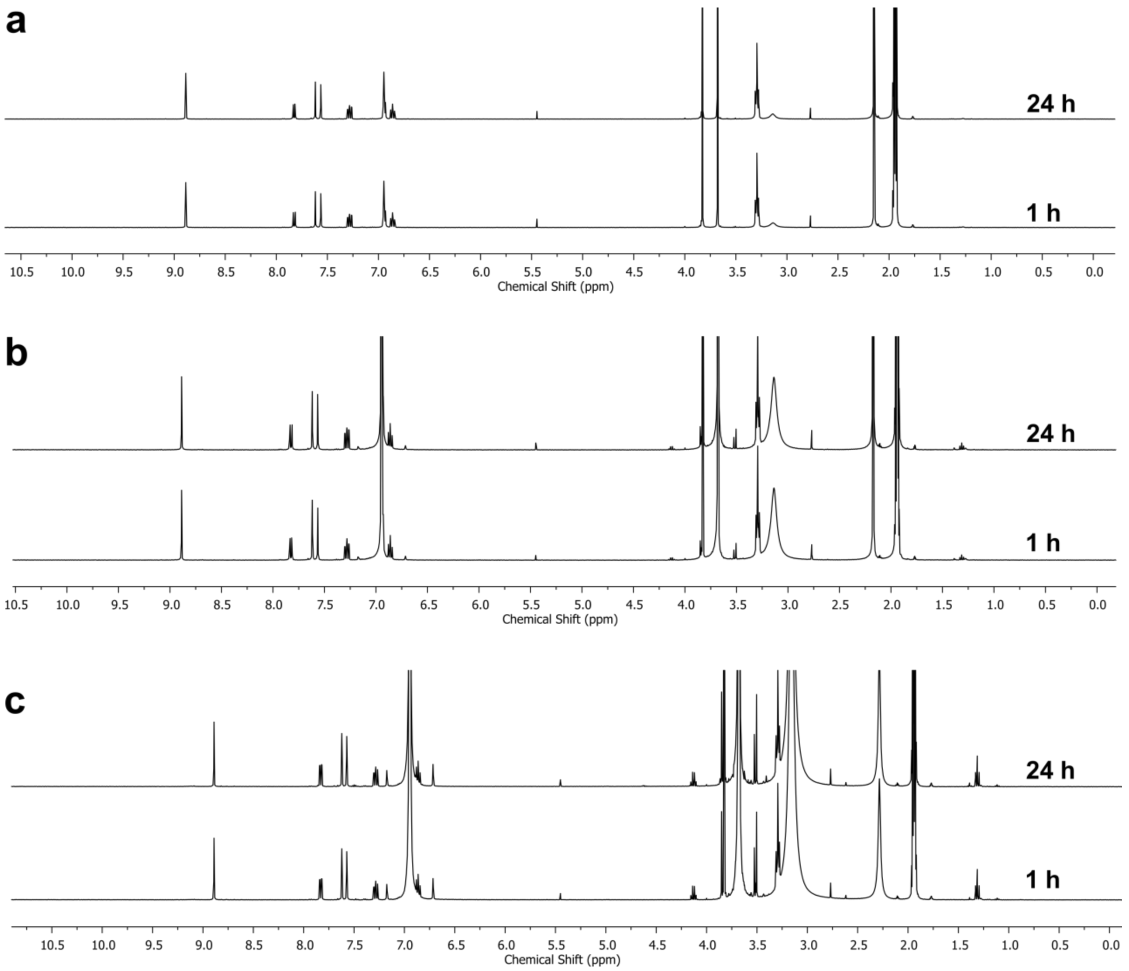

Figure S17. The ^{1}H NMR (400 Hz, 298 K, MeCN-d_3) of imine **1** (6 mM) and amine **A** at molar ratios of: (a) 1:1, (b) 1:10, and (c) 1:50, measured after 1 and 24 hours respectively.

5.1.2 From *Z*-1 to *E*-1, via Aminal-1

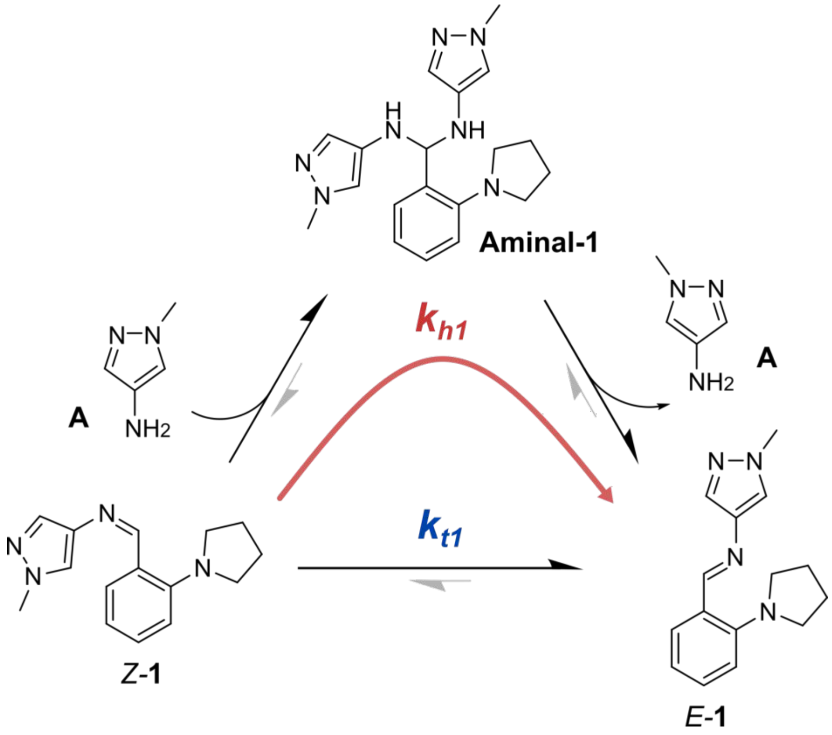

Figure S18. Schematic representation of Cycle 1 in the system. Here, k_{t1} corresponds to the unimolecular *Z*-to-*E* thermal isomerism; k_{h1} corresponds to the overall process through the aminal intermediate, not that while this is a reversible reaction, the conversion of *E*-**1** to **Aminal**-**1** and **Aminal**-**1** to *Z*-**1** are considered negligible.

To investigate the isomerization of *Z*-**1** in this cycle, a solution of imine **1** (6 mM in CD_3CN) was irradiated with 405 nm light for 1 hour to ensure the attainment of PSS. Different amounts of amine **A** were then immediately added, marking as the start time (t_0 = 0 min). The samples were kept in dark, and the progresses were monitored using ^{1}H NMR spectroscopy over time (Figure S19). The consumption of the *Z*-isomer can occur through both the thermal relaxation back to the *E*-isomer and isomerization via **Aminal-1** (Figure S18). The rate of *Z*-**1** consumption can therefore be described by the following equation:

$$\frac{d[Z-\mathbf{1}]}{dt} = -((k_{t1} \cdot [Z-\mathbf{1}]) + (k_{h1} \cdot [Z-\mathbf{1}] \cdot [\boldsymbol{A}]))$$

where k_{t1} is the rate constant of the thermal relaxation of *Z*-**1** (reported as 5.22 ×10^{-4} s^{-1} at 20 °C in our previous work[1]) and k_{h1} is the rate constant for the isomerization via **Aminal-1**. The concentration of *Z*-**1** and amine **A** can be determined from integration of the signals in ^{1}H NMR.

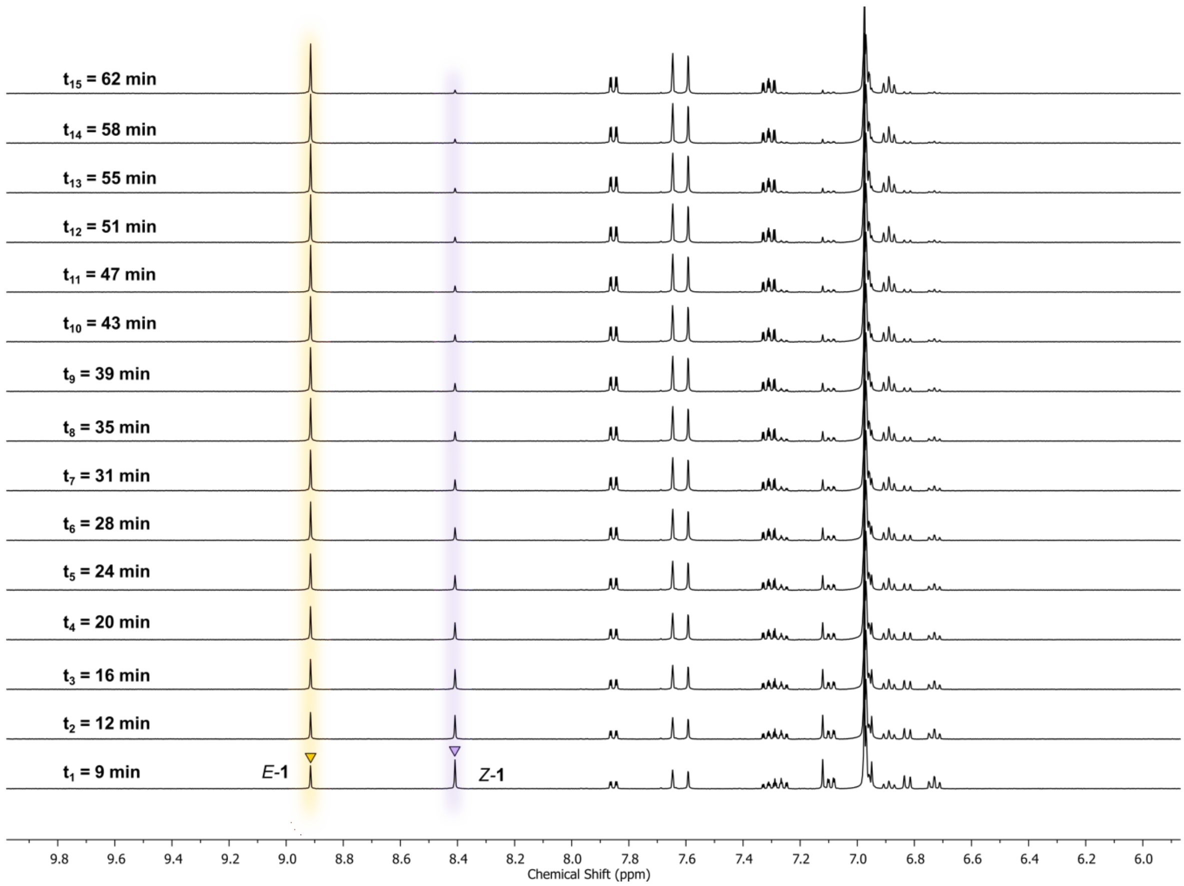

Figure S19. The ^{1}H NMR (400 Hz, 298 K, MeCN-*d₃*) of imine **1** (6 mM) following irradiation with 405 nm light for 1 hour and subsequent addition of 2 equivalents of amine **A**. The sample was kept in dark during measurement.

When plotting the rate for the isomerization via **Aminal-1** ($k_{h1} \cdot [Z - \mathbf{1}] \cdot [\boldsymbol{A}]$) against the concentration of *Z*-**1** as shown in Figure S20b, the k_{h1} can be obtained from the slope of the linear fit:

$$Slope = k_{h1} \cdot [\boldsymbol{A}] = 2.17 \times 10^{-4}$$

$$[\boldsymbol{A}] = 12.7\ mM$$

$$k_{h1} = 1.7 \times 10^{-2} M^{-1} s^{-1}$$

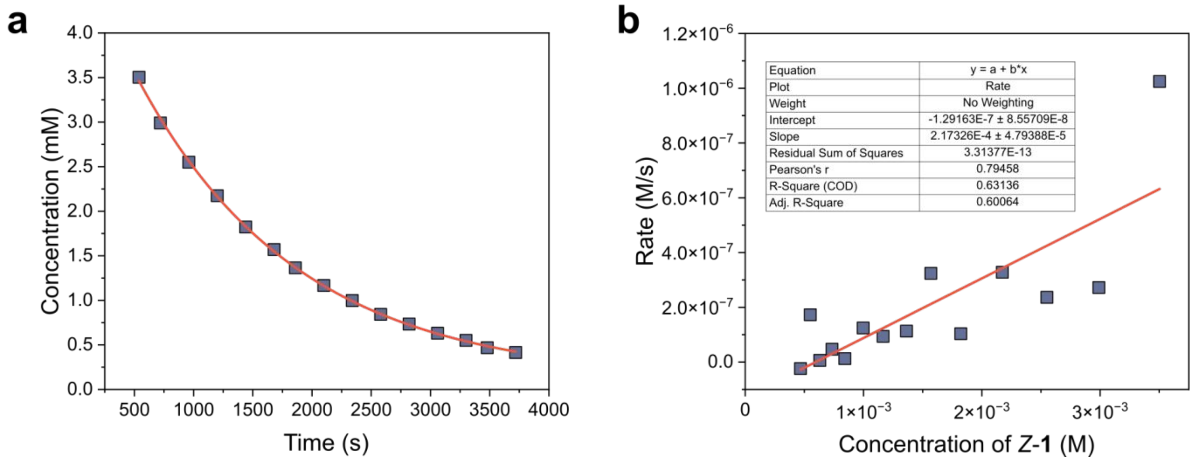

Figure S20. Kinetic profiles of the *Z*-**1** isomerization process with 2 equivalents amine **A**: **a,** Consumption of *Z*-**1** over time; **b,** Rates for the isomerization via **Aminal-1** at different time points plotted against the corresponding concentration of *Z*-**1** obtained from ^{1}H NMR.

Varying concentrations of amine A were used in parallel experiments (Figure S21), the corresponding rate constants k_{h1} are listed in Table S5, giving the average rate constant:

$$k_{h1} \approx 0.02\ \mathrm{M^{-1}s^{-1}}$$

Table S5. The rate constants (k_{h1}) obtained from the fittings with different concentrations of amine **A**.

	[**A**] / mM	k_{h1} / $\times 10^{-2}$ M^{-1} s^{-1}
Z-**1** + **A** (2 equiv.)	12.7	1.7 ± 0.4
Z-**1** + **A** (3 equiv.)	16.2	2.6 ± 0.2
Z-**1** + **A** (4 equiv.)	25.0	2.2 ± 0.4
Z-**1** + **A** (5 equiv.)	30.4	2.8 ± 0.2

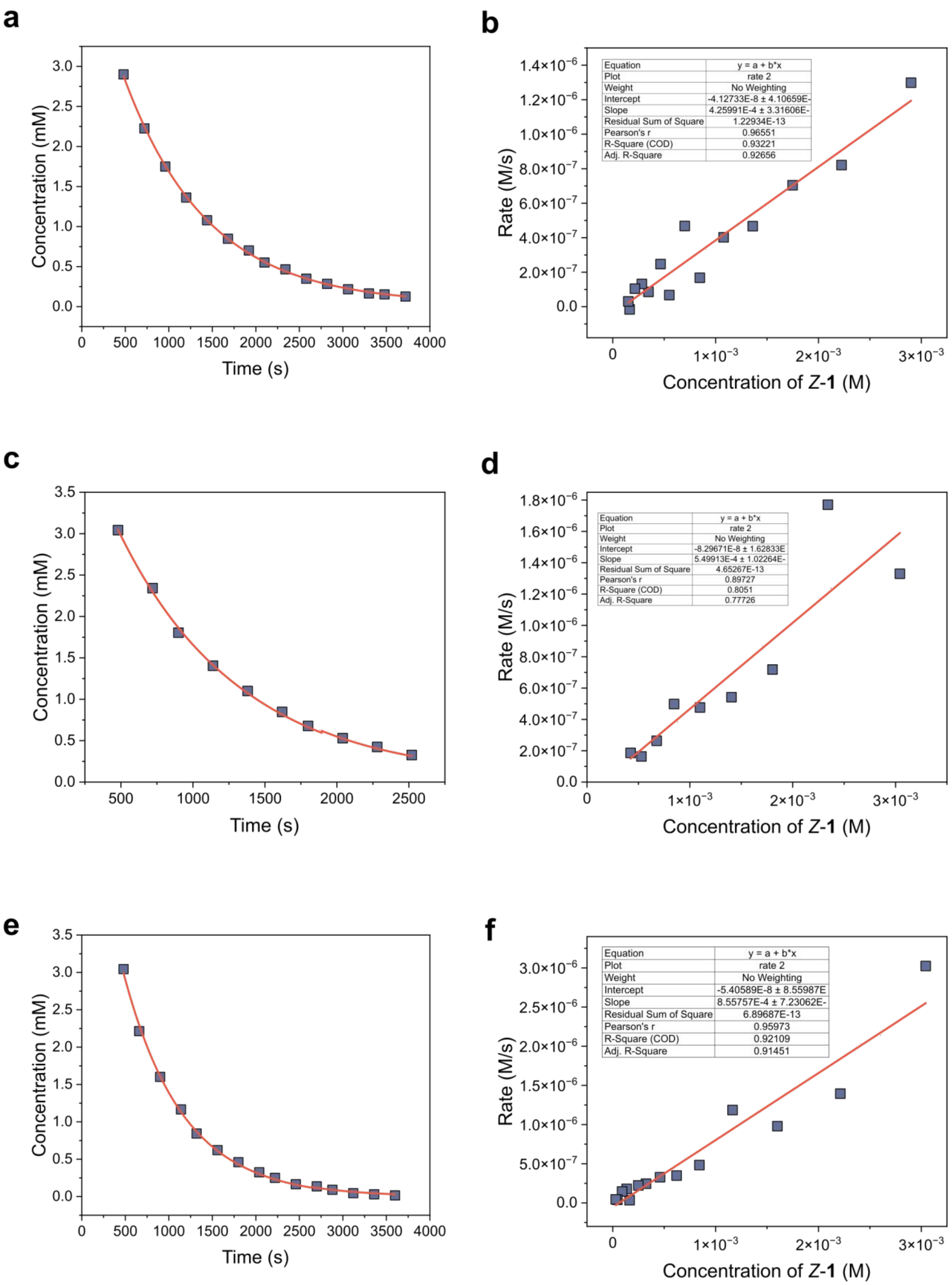

Figure S21. Kinetic profiles of the *Z*-**1** isomerization process with: **a-b**, 3 equivalents; **c-d**, 4 equivalents; **e-f**, 5 equivalents amine **A**.

5.2 Cycle 2: **Aminal-Mixed** involved isomerization and transimination from *Z*-**1**

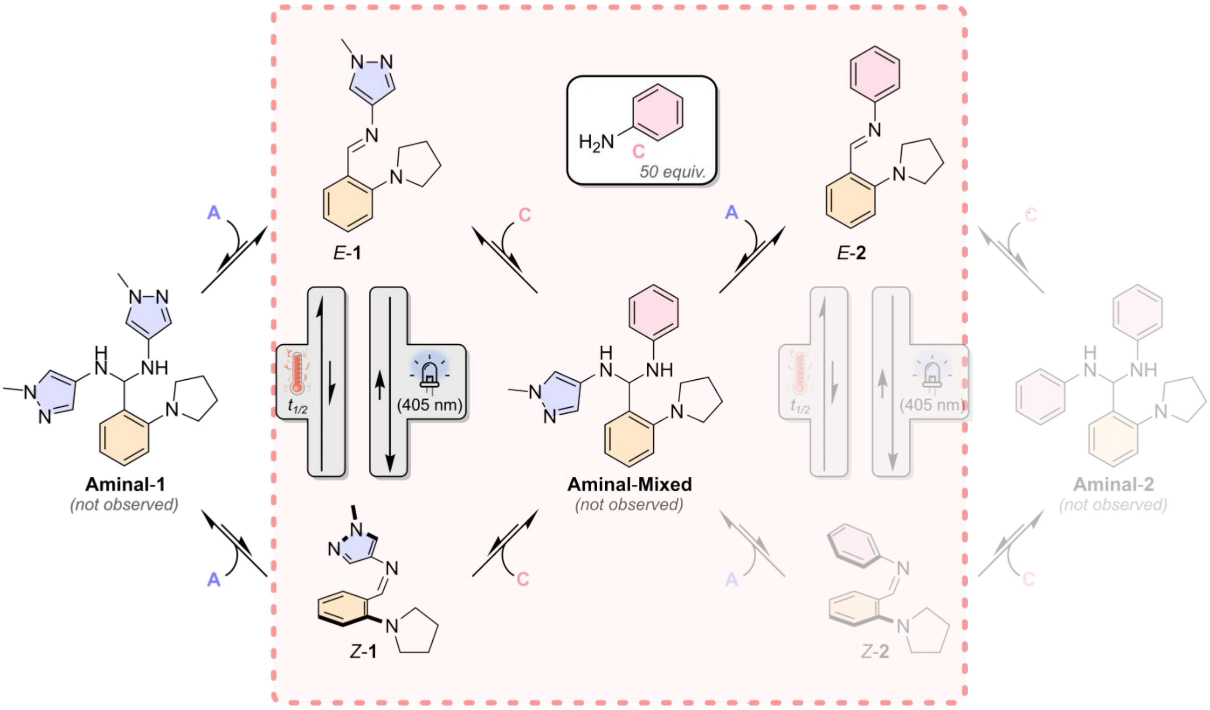

Figure S22. Schematic representation of the full system, highlighting Cycle 2. Grey shaded boxes indicate unimolecular reaction pathways.

To investigate the isomerization of *Z*-**1** via **Aminal-Mixed**, a solution of imine **1** (6 mM in CD_3CN) was irradiated with 405 nm light for 1 hour to ensure that the PSS has been reached. 50 equivalents of amine **C** were then immediately added, initiating the reaction (t_0 = 0 min). The samples were kept in dark, and the reaction progress was monitored overtime using ^1H NMR spectroscopy (Figure S23).

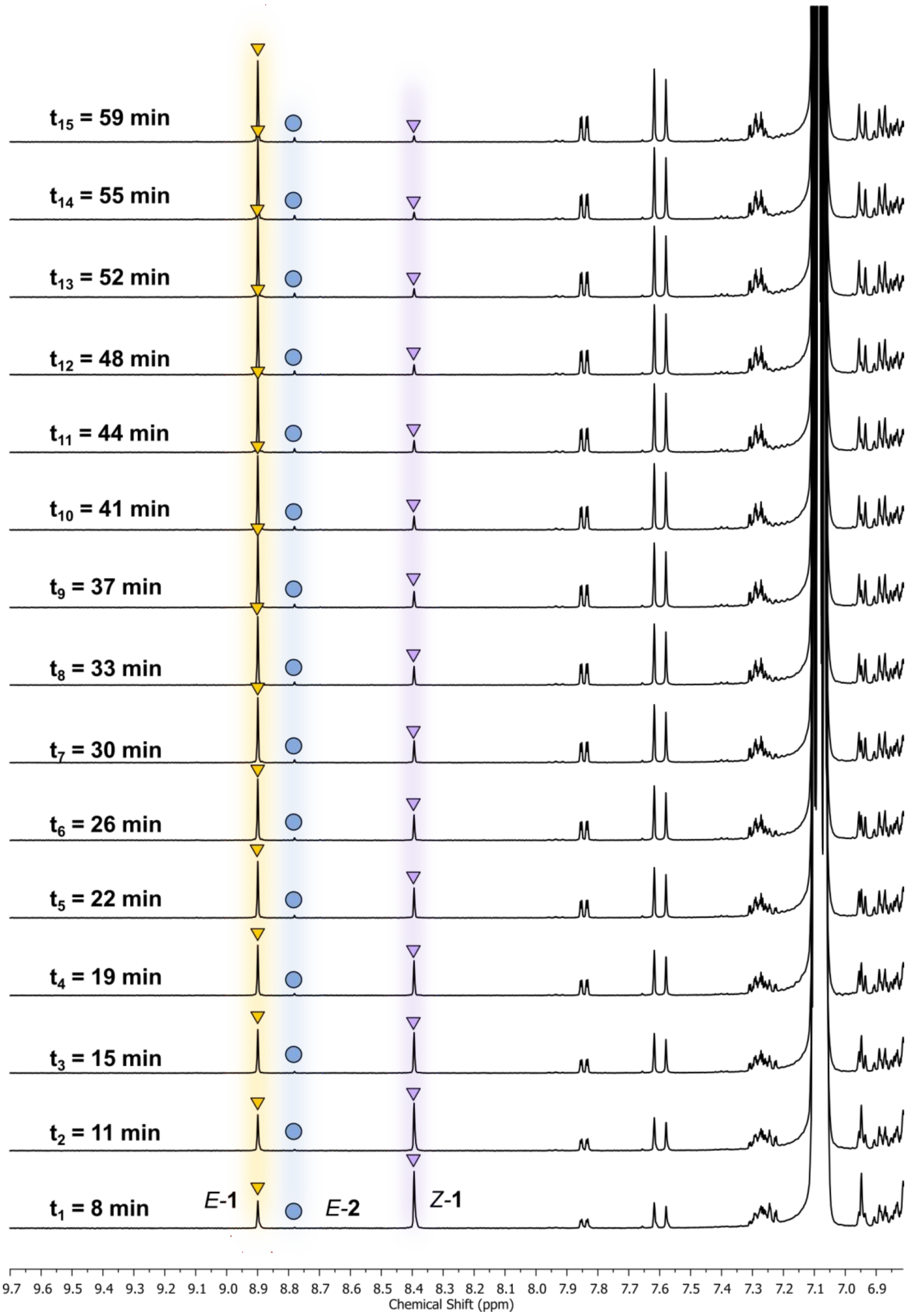

Figure S23. The ^{1}H NMR (400 Hz, 298 K, MeCN-d_3) of imine **1** (6 mM) following irradiation with 405 nm light for 1 hour and subsequent addition of 50 equivalents of aniline (**C**). The sample was kept in dark during measurement.

The *Z*-**1** species can be consumed through four pathways: unimolecular thermal isomerization back to *E*-**1**, two bimolecular aminal-mediated isomerizations (one with **Aminal-1** and one with **Aminal-Mixed**) back to *E*-**1**, and the bimolecular reaction of transimination with amine **C** to form *E*-**2**. The rate of *Z*-**1** consumption can be described by the following equation:

$$\frac{d[Z-\mathbf{1}]}{dt} = -((k_{t1} \cdot [Z-\mathbf{1}]) + (k_{m1} \cdot [Z-\mathbf{1}] \cdot [\boldsymbol{C}]) + (k_{h1} \cdot [Z-\mathbf{1}] \cdot [\boldsymbol{A}]) + (k_{2f} \cdot [Z-\mathbf{1}] \cdot [\boldsymbol{C}]))$$

where k_{t1} is the rate constant of the thermal relaxation of *Z*-**1**,[1] k_{m1} is the rate constant for the isomerization via **Aminal-Mixed** to afford *E*-**1**, k_{h1} is the rate constant for the isomerization via **Aminal-1** intermediates, and k_{2f} is the rate constant from *Z*-**1** to *E*-**1** via **Aminal-Mixed** (Section 4.2 of Supporting Information), as depicted in Figure S25. The concentrations of *Z*-**1** and amine **C** can be determined from the integration of the signals in the ^{1}H NMR spectra.

The formation of *E*-**1** can be attributed to thermal relaxation from *Z*-**1**, two aminal-mediated isomerizations, and the underlying equilibrium between *E*-**1** and *E*-**2**. The rate of *E*-**1** formation can be described by the following equation:

$$\frac{d[E-\mathbf{1}]}{dt} = (k_{t1} \cdot [Z-\mathbf{1}]) + (k_{m1} \cdot [Z-\mathbf{1}] \cdot [\boldsymbol{C}]) + (k_{h1} \cdot [Z-\mathbf{1}] \cdot [\boldsymbol{A}]) + (k_{1b} \cdot [E-\mathbf{2}] \cdot [\boldsymbol{A}]) - (k_{1f} \cdot [E-\mathbf{1}] \cdot [\boldsymbol{C}])$$

where k_{t1} is the rate constant of the thermal relaxation of *Z*-**1**,[1] k_{m1} is the rate constant for the isomerization via **Aminal-Mixed** intermediate, k_{h1} is the rate constant for the isomerization via **Aminal-1** intermediates, k_{1b} and k_{1f} are the composite rate constants between *E*-**1** to *E*-**2** (Section 4.1 of Supporting Information), as depicted in Figure S25. The concentrations of all species can be determined from ^{1}H NMR integrations.

The formation of *E*-**2** can be attributed to the transimination from *Z*-**1** and amine **C** via **Aminal-Mixed**, and the underlying equilibrium between *E*-**1** and *E*-**2**. The rate of *E*-**2** formation can be described by the following equation:

$$\frac{d[E-\mathbf{2}]}{dt} = (k_{2f} \cdot [Z-\mathbf{1}] \cdot [\boldsymbol{C}]) - (k_{1b} \cdot [E-\mathbf{2}] \cdot [\boldsymbol{A}]) + (k_{1f} \cdot [E-\mathbf{1}] \cdot [\boldsymbol{C}])$$

where k_{1b} and k_{1f} are the composite rate constants between *E*-**1** to *E*-**2** (Section 4.1 of Supporting Information), and k_{2f} is the rate constant from *Z*-**1** to *E*-**1** (Section 4.2 of Supporting Information), as depicted in Figure S25. The concentrations of all species can be determined from ^{1}H NMR integrations.

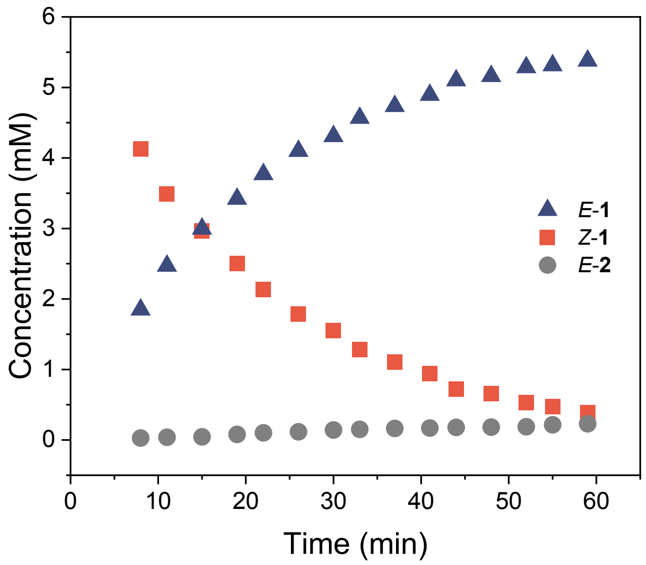

Figure S24. Concentration profiles of *E*-**1** (blue), *Z*-**1** (red), and *E*-**2** (grey) over time determined by integration of relative ^{1}H NMR signals and the initial concentration of imine **1** (6 mM).

Due to the high concentration (50 equivalents) of aniline (**C**), the variation of the aniline concentration throughout the experiment can be considered as negligible. Therefore, a similar approach to that described in Section 5.1.2 can be employed. By plotting the rate of *Z*-**1** isomerization to *E*-**1** via **Aminal-Mixed** ($k_{m1} \cdot [Z-\mathbf{1}] \cdot [\boldsymbol{C}]$) against the concentration of *Z*-**1**, the rate constant k_{m1} can be obtained:

$$[\boldsymbol{C}] = 3.6 \times 10^{-1} M$$

$$k_{m1} = 6.0 \times 10^{-4} M^{-1} s^{-1}$$

By analysing the combined data from cycles 1 and 2 (Figure S25), the ratio of *E*-**1** and *E*-**2** formation attributable to the aminal-mediated pathway can be obtained from the ratio of the corresponding rate constants k_{m1} and k_{2f}:

$$\frac{k_{m1}}{k_{m1} + k_{2f}} \times 100\% = \frac{6.0 \times 10^{-4}}{(6.0 + 3.8) \times 10^{-4}} \times 100\% \approx 60\%$$

$$1 - 60\% = 40\%$$

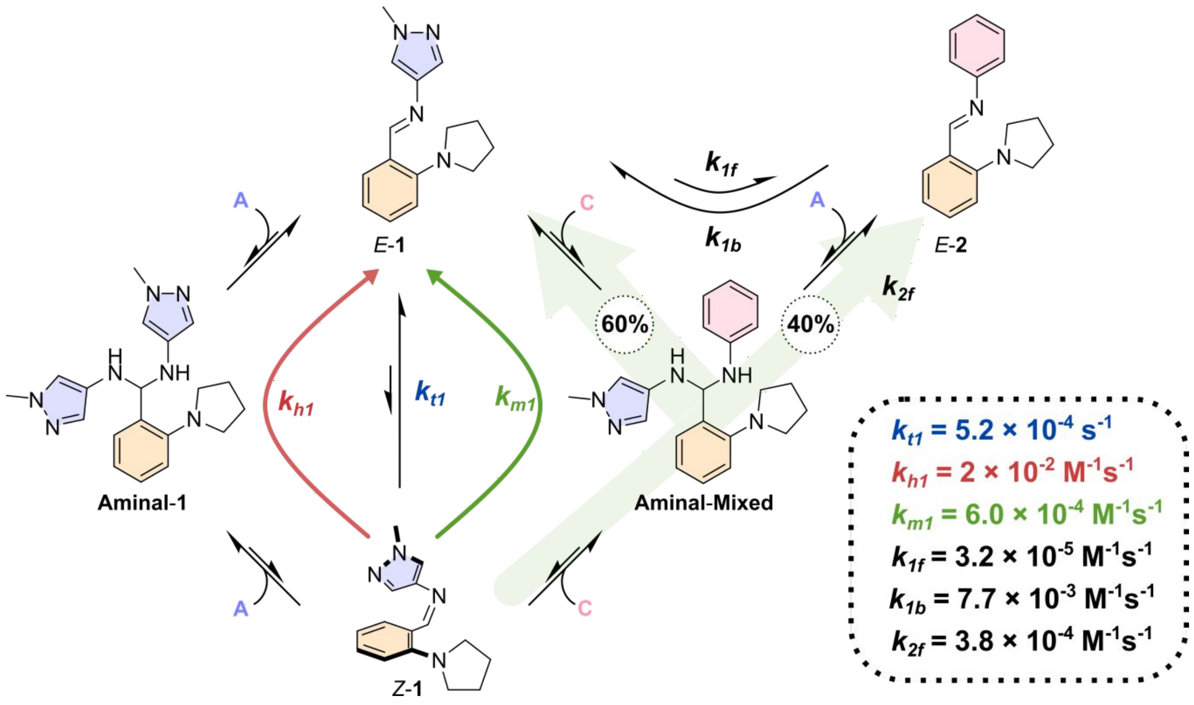

Figure S25. Schematic representation of the combined Cycle 1 and 2, illustrating all possible pathways, and accessible rate constants as listed in the dotted box. Note that k_{2f} corresponds to the pathway of *Z*-**1** to *E*-**2** via the green arrow. Here we have assumed the reverse thermal reactions from the *E*-isomers to the aminals and the *Z*-isomers to be negligible.

The observed concentration changes of imine **2** under different irradiation conditions are plotted together as shown in Figure S26, allowing for a comparison of the transimination reaction rates. In the sample which has been irradiated and then kept in the dark (green trace in Figure S26, the concentration of *Z*-**1** decreases over time due to isomerization, resulting in a change to a slower rate of transimination. While in the sample with continuous irradiation (red trace in Figure S26), the *Z*-**1** constantly replenished, providing more reactant to undergo transimination.

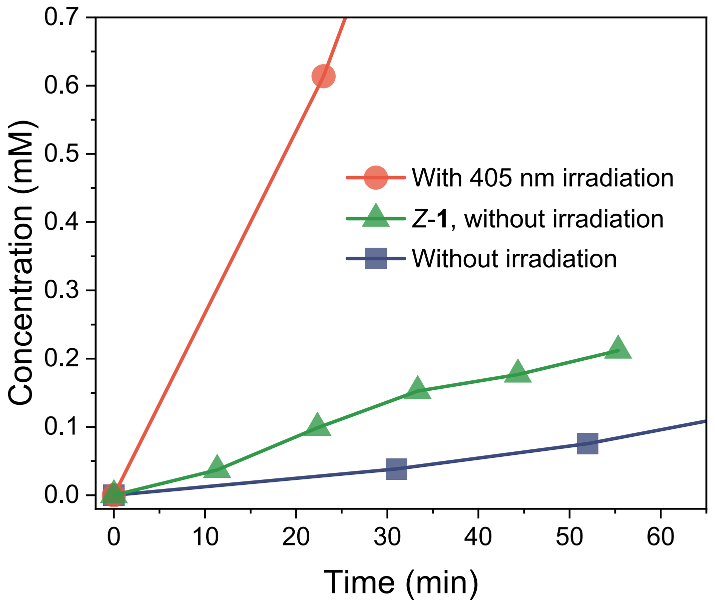

Figure S26. The observed concentration changes of imine **2** over time (up to 1 hour) under different irradiation conditions. The data were obtained using ^{1}H NMR spectroscopy (initial concentration of imine **1** is 6 mM in CD_3CN). The curves represent the following conditions: red – with 405 nm irradiation (Figure S14); green – irradiated with 405 nm light and then kept in the dark during measurements (Figure S23); blue – no irradiation (Figure S11).

5.3 Cycle 3: Homo-aminal (**Aminal-2**) involved isomerization between *E*-**2** and *Z*-**2**

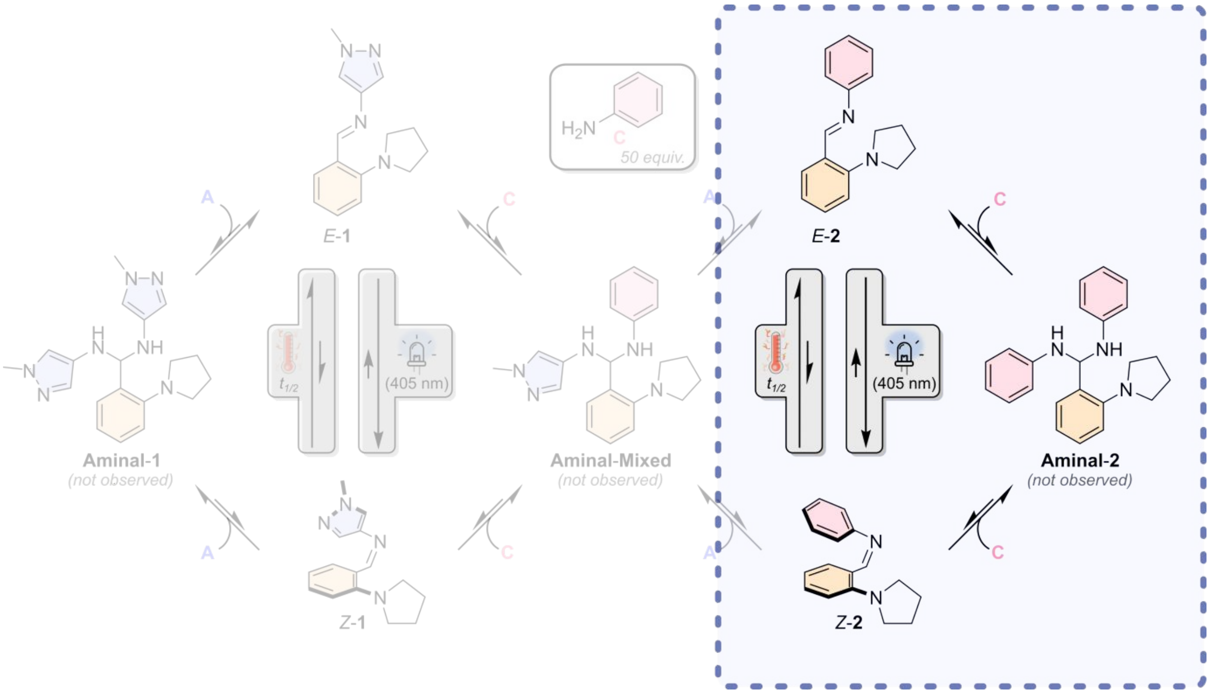

Figure S27. Schematic representation of the full system, highlighting Cycle 3. Grey shaded boxes indicate unimolecular reaction pathways.

Similar to the cycle 1, the photoisomerization from *E*-**2** to *Z*-**2** and the thermally relax back from *Z*-**2** to *E*-**2** are both monomolecular processes, and the presence of excess amine can lead to aminal formation. To investigate the potential impact of **Aminal-2** formation, we performed control experiments by adding aniline (**C**) into the solution of imine **2**.

5.3.1 From *E*-2 to Aminal-2

To investigate the influence of **Aminal-2** on the *E*-**2**/*Z*-**2** isomerization, a large amount of amine **C** was added in the solution of imine **2** at molar ratios of (a) 1:50, (b) 1:100, and (c) 1:200 as shown in Figure S28. ^{1}H NMR analysis after 1 and 24 hours revealed no signals corresponding to **Aminal-2**, suggesting both slow formation and rapid consumption of the aminal intermediate. Additionally, no observation of the *Z*-**2** isomer was made, indicating a strong thermodynamic preference for forming *E*-**2**.

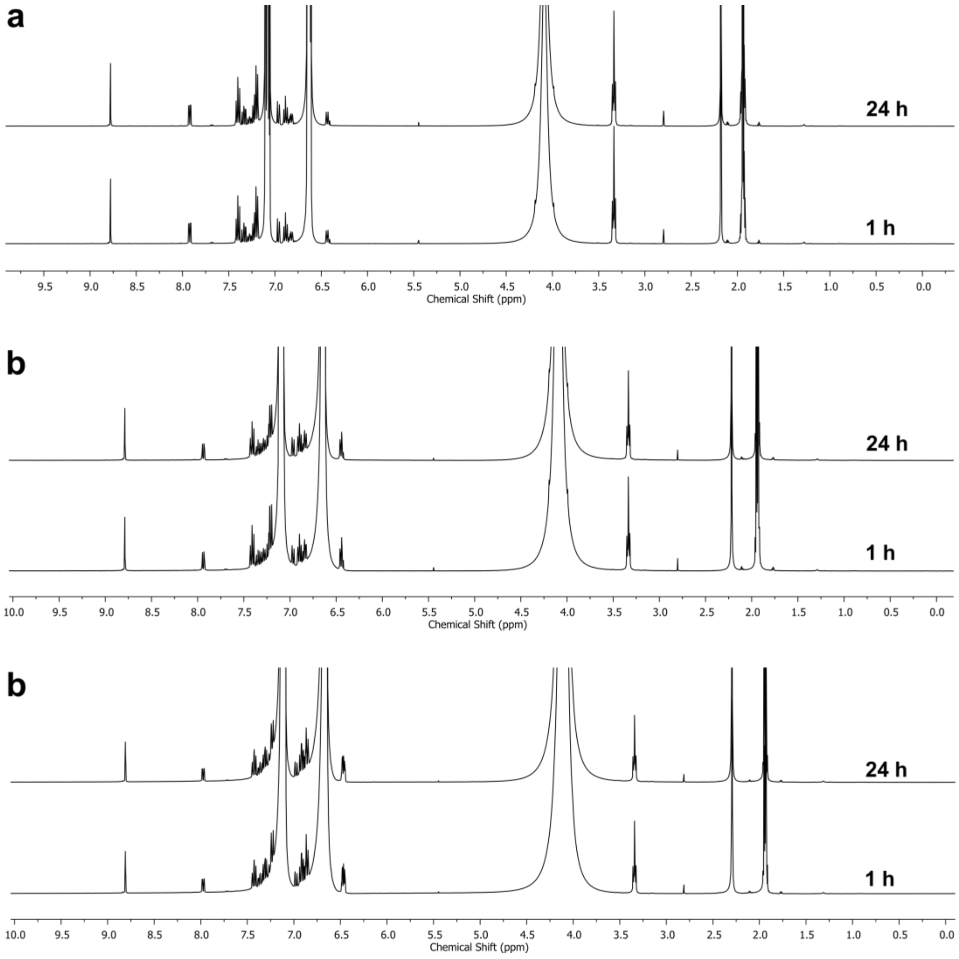

Figure S28. The ^{1}H NMR (400 Hz, 298 K, MeCN-d_3) of imine **2** (6 mM) and amine **C** at molar ratios of: (a) 1:50, (b) 1:100, and (c) 1:200, measured after 1 and 24 hours respectively.

5.3.2 From *Z*-2 to Aminal-2, to *E*-2

Due to the short thermal half-life of imine **2** (< 3 seconds, Section 3.2, Supporting Information), monitoring concentration changes of reaction by ^{1}H NMR spectroscopy is not feasible. Therefore, UV/vis spectroscopy was employed as an alternative approach to investigate the isomerization behaviour of imine **2** under conditions analogous to NESS in Section 4.2. Solutions of imine **2** (2.5 mM) in MeCN were prepared for measurements on the diode array setup (Section 1.2.1, Supporting Information), using a 1 mm cuvette. The reaction progress was monitored by UV/vis spectroscopy at 410 nm, a wavelength where imine **2** has absorbance around 1 (Figure S29).

To investigate the influence of aniline on the isomerization of imine **2**, a mixture of imine **2** (2.5 mM) and aniline (0.3 M) was prepared in MeCN, mimicking the concentration used in the NESS sample (Section 4.2). The reaction progress was monitored by UV/vis spectroscopy at 410 nm, a wavelength where imine **2** has absorbance around 1. Since aniline exhibits negligible absorbance above 350 nm, this choice of monitoring wavelength also minimizes interference from the constant background signal of aniline, allowing for a clearer observation of changes in absorbance due to imine **2** alone (Figure S30). By plotting the absorbance at 410 nm against time, the exponential profiles were observed, suggesting that the isomerization from *Z*-**2** to *E*-**2** is dominated by the unimolecular thermal relaxation, thus the exponential fitting was still applied to determine the approximate rate constants here.

As shown in Table S6, the rate constants for the overall isomerization of imine **2** at 20 °C under different conditions are comparable, suggesting a negligible contribution from the aminal-mediated isomerization pathway in this step.

Table S6. The rate constants (*k*) for the isomerization of imine **2** at 20 °C under various conditions, including the presence/absence of aniline (**C**).

	k at 20 °C (s^{-1})	$t_{1/2}$ at 20 °C (s)
Imine **2** (70 µM)	2.6×10^{-1}	2.7
Imine **2** (2.5 mM)	2.6×10^{-1}	2.6
Imine **2** (2.5 mM) + amine **C** (0.3 M)	1.9×10^{-1}	3.6

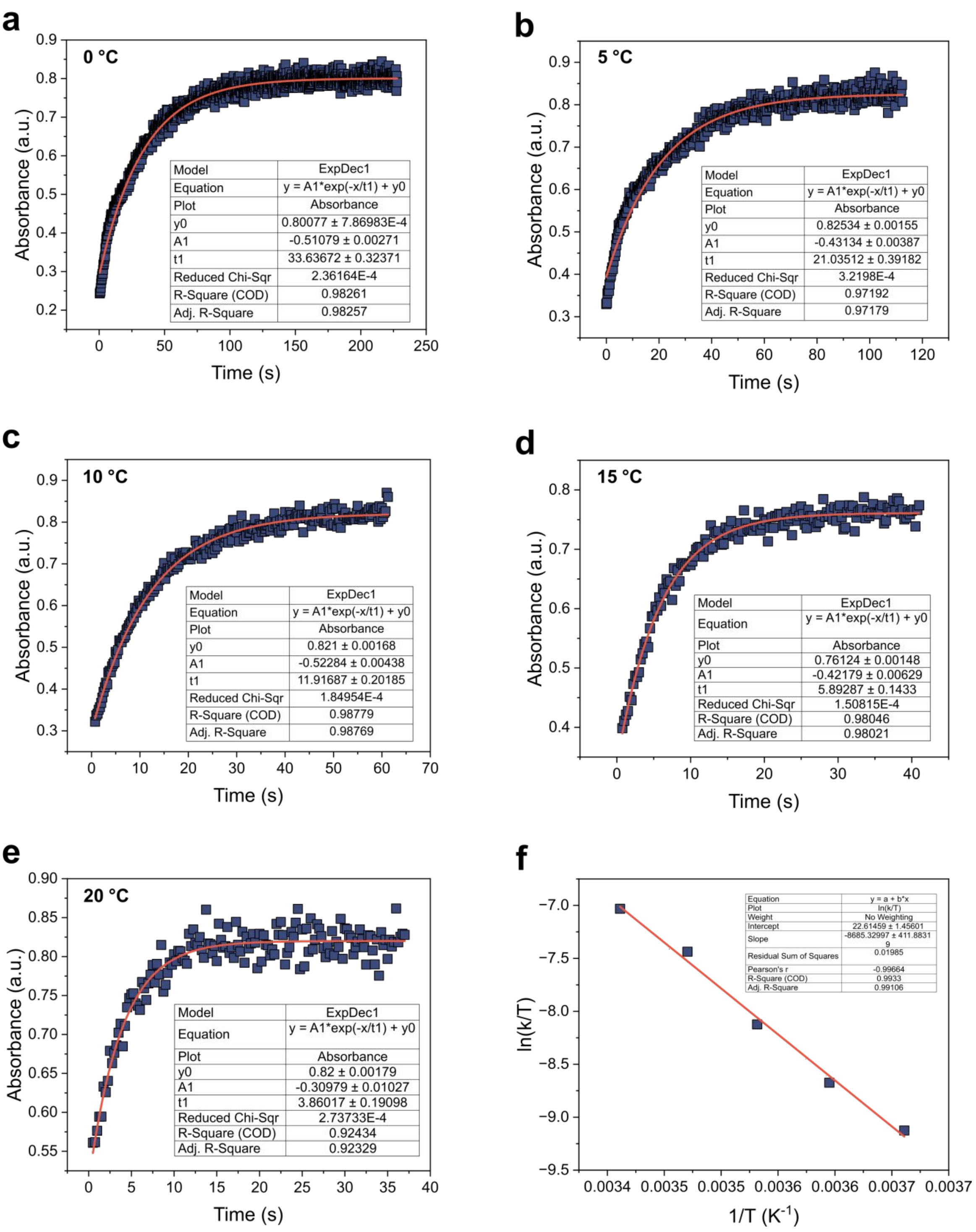

Figure S29. The change in absorbance of **2** (2.5 mM in MeCN) at 410 nm over time at **a**, 0 °C, **b**, 5 °C, **c**, 10 °C, **d**, 15 °C, **e**, 20 °C, with the increase in absorbance relating to the thermal isomerization of the *Z*-isomer to the *E*-isomer. The sample was irradiated under 405 nm LED for 2 minutes in acetonitrile before the measurement started. The exponential fittings were applied to determine the rate constants and thus thermal half-lives. **f,** The Eyring plot of **2** was generated using the rate constants calculated at different temperatures.

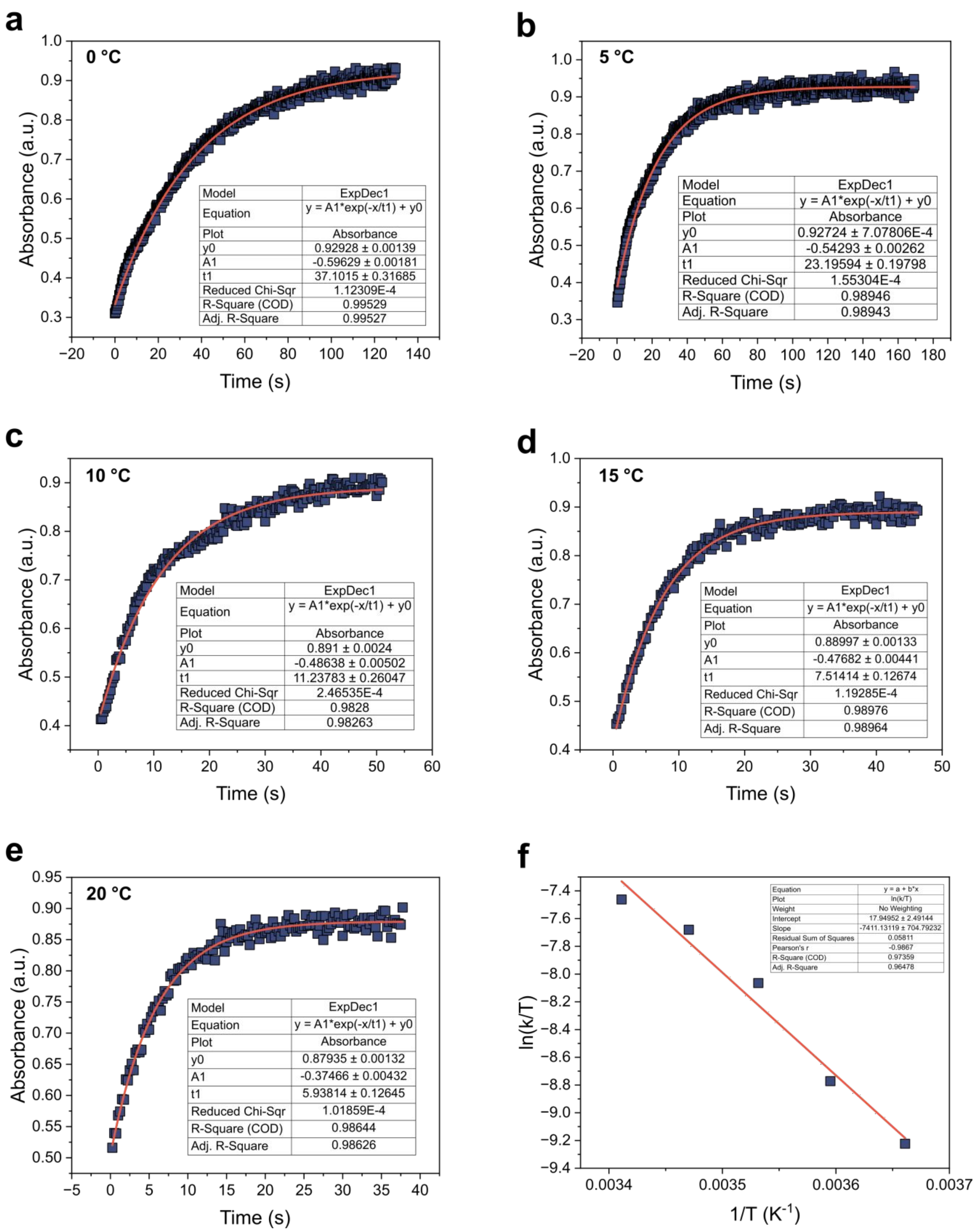

Figure S30. The change in absorbance of **2** (2.5 mM in MeCN) with aniline (0.3 M in MeCN) at 410 nm over time at **a**, 0 °C, **b**, 5 °C, **c**, 10 °C, **d**, 15 °C, **e**, 20 °C, with the increase in absorbance relating to the thermal isomerization of the *Z*-isomer to the *E*-isomer. The sample was irradiated under 405 nm LED for 2 minutes in acetonitrile before the measurement started. The exponential fittings were applied to determine the rate constants and thus thermal half-lives. **f,** The Eyring plot of **2** was generated using the rate constants calculated at different temperatures.

5.4 Cycle 4: Mixed-aminal involved isomerization and transimination from *Z*-2

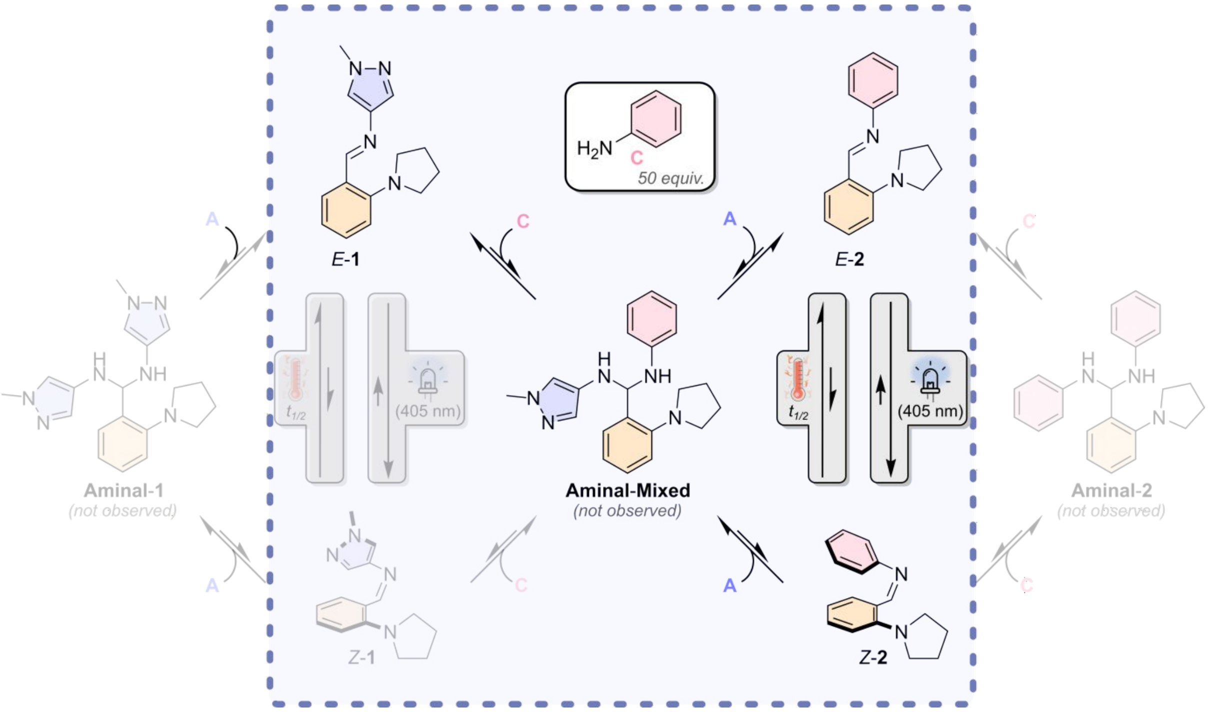

Figure S31. Schematic representation of the full system, highlighting Cycle 4. Grey shaded boxes indicate unimolecular reaction pathways.

The rate constant for the pathway from *Z*-**2** to *E*-**1** via **Aminal-Mixed** (k_{2b}) was determined in Section 4.2 of Supporting Information. Given that the ratio of *E*-**1** and *E*-**2** formation attributable to the aminal-mediated pathway is approximately 60% to 40% (Figure S25), the rate constant for the pathway from *Z*-**2** to *E*-**2** via **Aminal-Mixed** (k_{m2}) can be approximated by considering the relative contributions of each pathway to the overall formation of *E*-**1** and *E*-**2**:

$$k_{2b} = 1.6 \times 10^{-1} M^{-1}\, s^{-1}$$

$$\frac{k_{m2}}{k_{2b}} = \frac{40\%}{60\%}$$

$$k_{m2} = 1.1 \times 10^{-1} M^{-1}\, s^{-1}$$

5.5 Description of the Full System

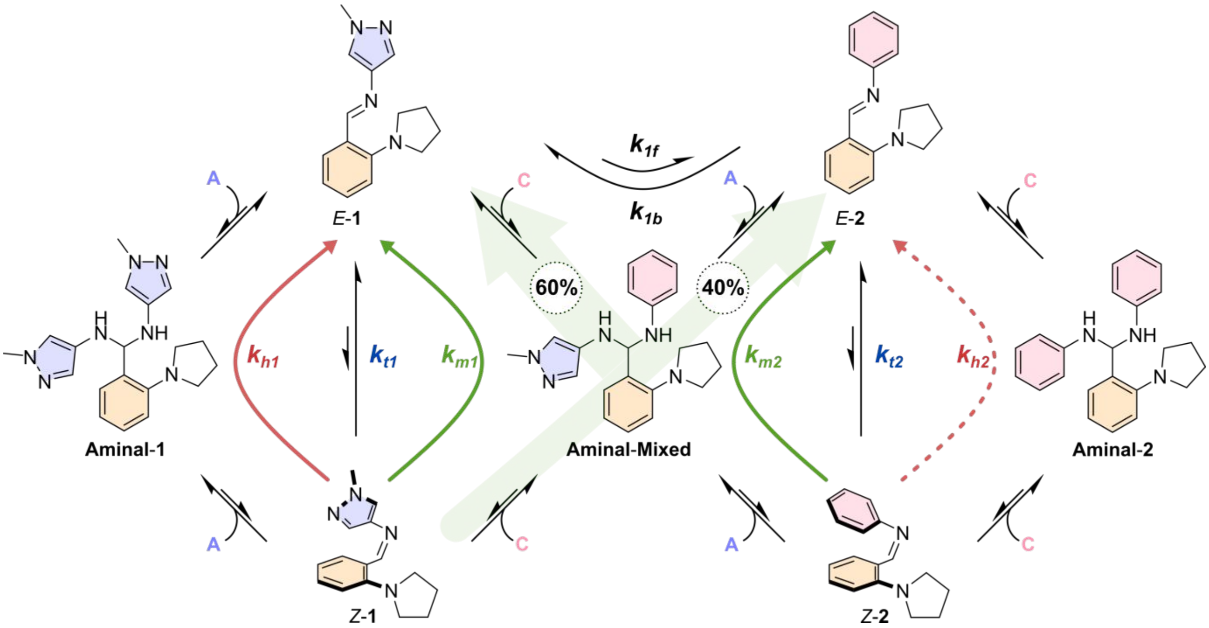

Figure S32. Schematic representation of the system with all possible pathways. Here we have assumed the reactions from the *E*-isomers to the aminals and the *Z*-isomers to be negligible. Note that under constant irradiation, 95% of **1** exists as the *Z*-isomer, 5% as the *E*-isomer and 30% of **2** exists as *Z*-isomer and 70% of **2** as *E*-isomer.

In conclusion, the rate constants for all possible isomerization pathways of *Z*-**1** to *E*-**1** and *Z*-**2** to *E*-**2** are summarized in Tables S7 and S8, respectively.

Table S7. The rate constants for all possible isomerization pathways of *Z*-**1** to *E*-**1**.

Pathway	Rate Constants
Thermal relaxation from *Z*-**1** to *E*-**1**	$k_{t1} = 5.2 \times 10^{-4} s^{-1}$
Z-**1** → **Aminal-1** → *E*-**1**	$k_{h1} = 2 \times 10^{-2} M^{-1} s^{-1}$
Z-**1** → **Aminal-Mixed** → *E*-**1**	$k_{m1} = 6.0 \times 10^{-4} M^{-1} s^{-1}$

Table S8. The rate constants for all possible isomerization pathways of *Z*-**2** to *E*-**2**.

Pathway	Rate Constants
Thermal relaxation from *Z*-**2** to *E*-**2**	$k_{t2} = 2.6 \times 10^{-1} s^{-1}$
Z-**2** → **Aminal-2** → *E*-**2**	$Negligible$
Z-**2**→ **Aminal-Mixed** → *E*-**2**	$k_{m2} = 1.1 \times 10^{-1} M^{-1}\ s^{-1}$

Continuous irradiation efficiently drives imine **1** to its PSS within a short time frame (Figure S9, Section 3.4, Supporting Information). This rapid photodynamic process between *E*-**1** and *Z*-**1** isomers outcompetes any thermodynamic isomerization processes under these conditions. In contrast, due to the fast thermal relaxation of imine **2** from the *Z*-isomer to the *E*-isomer at room temperature, the competition between the photodynamic process and the thermal relaxation in imine **2** results in a steady-state under continuous irradiation, with approximately 30% of **2** existing as the *Z*-isomer (Figure S9, Section 3.4, Supporting Information).

Due to the negligible contributions of the pathways involving **Aminal-1** and **Aminal-2** under continuous irradiation, the rate constants for the isomerization and transimination pathways involving only the **Aminal-Mixed** intermediate were summarized in Table S9.

Table S9. The rate constants for all possible isomerization and transimination pathways involving **Aminal-Mixed** intermediate.

Pathway	Rate Constants
E-**1** → **Aminal-Mixed** → *E*-**2**	$k_{1f} = 3.2 \times 10^{-5} M^{-1} s^{-1}$
E-**2** → **Aminal-Mixed** → *E*-**1**	$k_{1b} = 7.7 \times 10^{-3} M^{-1} s^{-1}$
Z-**1** → **Aminal-Mixed** → *E*-**1**	$k_{m1} = 6.0 \times 10^{-4} M^{-1} s^{-1}$
Z-**1** → **Aminal-Mixed** → *E*-**2**	$k_{2f} = 3.8 \times 10^{-4} M^{-1} s^{-1}$
Z-**2** → **Aminal-Mixed** → *E*-**2**	$k_{m2} = 1.1 \times 10^{-1} M^{-1} s^{-1}$
Z-**2** → **Aminal-Mixed** → *E*-**1**	$k_{2b} = 1.6 \times 10^{-1} M^{-1} s^{-1}$

In general, under continuous irradiation, a directional photoswitching behaviour is observed. The *E*-**1** isomers are efficiently converted to *Z*-**1** via the photodynamic process to achieve the PSS (95% of **1** as the *Z*-isomer). This continuous generation of *Z*-**1** isomers drives the subsequent formation of *E*-**2** through the **Aminal-Mixed** pathway (40%), and the *E*-**1** generated through this **Aminal-Mixed** pathway (60%) can be efficiently converted to *Z*-**1** again, resulting in further formation of *E*-**2**. At the same time, *E*-**2** undergoes similar processes to form *E*-**1**, but in a rather low efficiency due to its poor photoswitching behaviour (30% of **2** as

the *Z*-isomer, compared to 95% of imine **1** as the Z-isomer under the same conditions). This competition between more efficient conversion (*E*-**1**→*Z*-**1**→*E*-**2**) and less efficient conversion (*E*-**2**→*Z*-**2**→*E*-**1**) leads to a net accumulation of *E*-**2**, resulting in the directional property of the system under irradiation.

5.6 Flux of the Cycles

For the anticlockwise cycle (ACWC, i.e., *E*-**1** to *Z*-**1** to *E*-**2** to *E*-**1**) and clockwise cycle (CWC, i.e., *E*-**2** to *Z*-**2** to *E*-**1** to *E*-**2**), the ratcheting constants can be calculated using the rate constants of each reversible step:

For the ACWC:

$$Ratcheting\ Constant, K_{r1} = \frac{k_{(\mathbf{1},\,PSS+)} \times k_{2f} \times k_{1b}}{k_{(\mathbf{1},PSS-)} \times k_{1f} \times k_{(E-\mathbf{2}\ to\ Z-\mathbf{1})}}$$

For the CWC:

$$Ratcheting\ Constant, K_{r2} = \frac{k_{(\mathbf{2},\,PSS+)} \times k_{2b} \times k_{1f}}{k_{(\mathbf{2},PSS-)} \times k_{1b} \times k_{(E-\mathbf{1}\ to\ Z-\mathbf{2})}}$$

However, given that *E*-**2** to *Z*-**1** and *E*-**1** to *Z*-**2** are highly unfavoured, we could not probe these rate constants experimentally. We can infer that these rate constants will be small, thus resulting in a large ratcheting constant, and we assume that both cycles can be approximated to being unidirectional.

Considering the contribution of each cycle to the overall operation of the system, we define the ratio of the two ratcheting constants as, K_s,:

$$K_S = \frac{K_{r1}}{K_{r2}} = \frac{\dfrac{0.95 \times k_{2f} \times k_{1b}}{0.05 \times k_{1f} \times k_{(E-\mathbf{2}\ to\ Z-\mathbf{1})}}}{\dfrac{0.3 \times k_{2b} \times k_{1f}}{0.7 \times k_{1b} \times k_{(E-\mathbf{1}\ to\ Z-\mathbf{2})}}}$$

$$K_S = 6.1 \times 10^3 \times Q\ ,\ where\ Q = \frac{k_{(E-\mathbf{1}\ to\ Z-\mathbf{2})}}{k_{(E-\mathbf{2}\ to\ Z-\mathbf{1})}}$$

Thus, a net ACWC is achieved ($K_s > 1$) when Q > 1.6×10^{-4}, otherwise a net CWC is present.

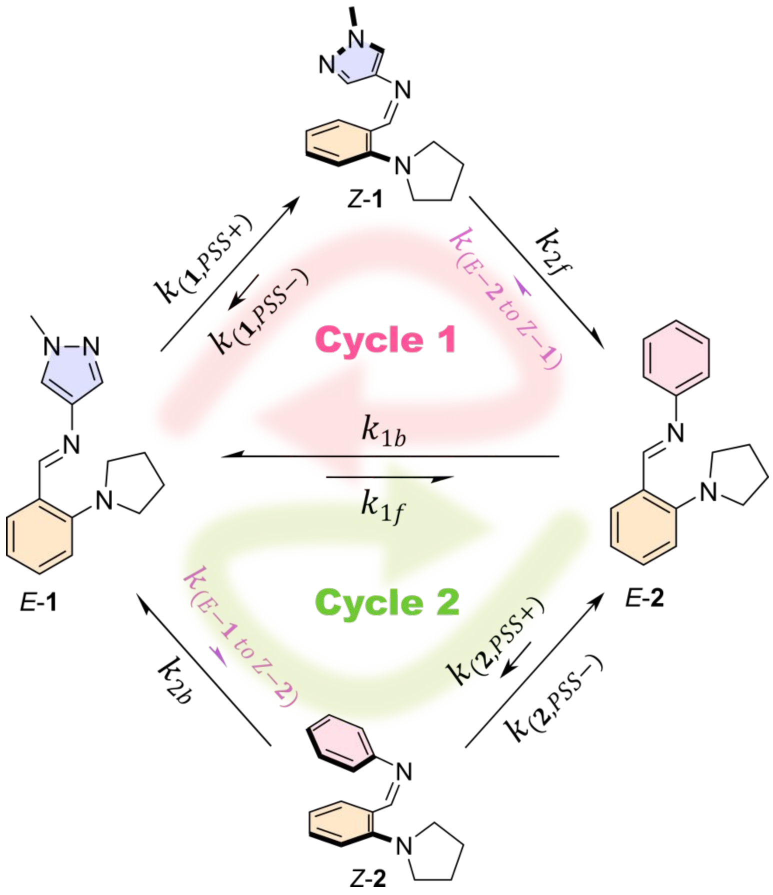

Figure S33. An alternative representation of Figure 2 shown in the main manuscript where the system is shown as two triangular cycles that makes up a diamond, as opposed to the square cycle of Figure 2. In this representation, the anticlockwise cycle (ACWC) and clockwise cycle (CWC) nomenclature used in the main text to describe Figure 2 is not valid. For this diamond representation, the cycle 1 results in a net flux of imine **1** to imine **2** via the photoisomerism pathway while cycle 2 shows a net flux of imine **2** to imine **1**. The analysis above in this section is still value for this approach by replacing the ACWC with cycle 1 and CWC with cycle 2, respectively.

6. Energy Storage

In the system, there are two NESS states of interest. The photoisomerism inherently creates a NESS between the *E* and *Z*-isomers and there is also a NESS linked to the dynamic-covalent systems constitution. Given that the *Z*-isomer is metastable, this photodynamic equilibrium can also store energy.

To determine the energy stored in the metastable *Z*-isomers, the energy difference between the *E*- and *Z*-isomers needs to be determined ($\Delta G_{Z\text{-}E}$). This can be done computationally (see Section 7 of the Supporting Information). Knowing the overall concentration of the system, the proportions of the two imines generated at the NESS of the system (i.e. that shown in Figure 3a of the main manuscript) and the proportion of each imine that exists as the *Z*-isomer, allows the concentration of *Z*-**1** and *Z*-**2** in the NESS system to be determined. The product of this concentration with the $\Delta G_{Z\text{-}E}$ affords the amount of energy stored in the *Z*-isomers at the NESS.

The energy stored in the non-equilibrium distribution of the imines can be calculated based on the concentrations of each species at equilibrium and the non-equilibrium state. This approach reflects how much each species deviates from its equilibrium concentration, contributing to the overall free energy storage. The total stored free energy is obtained by summing the contributions from all individual species as described in the following equation:[7,8]

$$stored\ free\ energy = RT \sum_i ([i] ln \frac{[i]}{[i]_{eq}} - [i] + [i]_{eq})$$

In this equation, $[i]_{eq}$ represents the equilibrium concentration of each species, and $[i]$ represents its concentration in the non-equilibrium state. Since the concentrations are used here to determine the number of molecules involved, the unit of this equation is energy per volume.

In our system, the sample reached NESS upon irradiation with 405 nm light (Section 4.2), compared to the thermally equilibrated sample (Section 4.1), the stored free energy in the solution (6 mM in MeCN) was found to be 0.33 J L^{-1}. This value is in agreement with other reported systems in literature.[8]

7. Computational Studies

The DFT approach we adopted was based on our previously reported approach.[1] Initial guess geometries of the *E*-isomer and *Z*-isomer of imine **2** were constructed in Avogadro (version 1.2.0) and were geometry-optimized using an MMFF94s force field.[9] All DFT calculations were performed on the lowest energy conformer using ORCA (version 5.0.4) software.[10–12] The geometries of the lowest energy *E* and *Z* conformers were further optimised at ωB97X-D4/def2-TZVPP level of theory[13–18] with a CPCM solvation model for acetonitrile.[19] Ground-state equilibrium geometries of the *E* and *Z* isomers were confirmed by the absence of imaginary vibrational frequencies.

References

1 J. Wu, L. Kreimendahl, S. Tao, O. Anhalt and J. L. Greenfield, *Chem. Sci.*, 2024, **15**, 3872–3878.

2 International Union of Pure and Applied Chemistry (IUPAC), .

3 H. Eyring, *J. Chem. Phys.*, 1935, **3**, 107–115.

4 E. Fischer, *J. Phys. Chem.*, 1967, **71**, 3704–3706.

5 C. G. Hatchard, C. A. Parker and E. J. Bowen, *Proc. R. Soc. Lond. Ser. Math. Phys. Sci.*, 1956, **235**, 518–536.

6 K. Stranius and K. Börjesson, *Sci. Rep.*, 2017, **7**, 41145.

7 T. Sangchai, S. Al Shehimy, E. Penocchio and G. Ragazzon, *Angew. Chem. Int. Ed.*, 2023, **62**, e202309501.

8 F. Avanzini, E. Penocchio, G. Falasco and M. Esposito, *J. Chem. Phys.*, 2021, **154**, 094114.

9 M. D. Hanwell, D. E. Curtis, D. C. Lonie, T. Vandermeersch, E. Zurek and G. R. Hutchison, *J. Cheminformatics*, 2012, **4**, 17.

10 F. Neese, *WIREs Comput. Mol. Sci.*, 2012, **2**, 73–78.

11 F. Neese, *WIREs Comput. Mol. Sci.*, 2018, **8**, e1327.

12 F. Neese, F. Wennmohs, U. Becker and C. Riplinger, *J. Chem. Phys.*, 2020, **152**, 224108.

13 A. Najibi and L. Goerigk, *J. Comput. Chem.*, 2020, **41**, 2562–2572.

14 E. Caldeweyher, C. Bannwarth and S. Grimme, *J. Chem. Phys.*, 2017, **147**, 034112.

15 E. Caldeweyher, S. Ehlert, A. Hansen, H. Neugebauer, S. Spicher, C. Bannwarth and S. Grimme, *J. Chem. Phys.*, 2019, **150**, 154122.

16 E. Caldeweyher, J.-M. Mewes, S. Ehlert and S. Grimme, *Phys. Chem. Chem. Phys.*, 2020, **22**, 8499–8512.

17 F. Weigend and R. Ahlrichs, *Phys. Chem. Chem. Phys.*, 2005, **7**, 3297.

18 F. Weigend, *Phys. Chem. Chem. Phys.*, 2006, **8**, 1057.

19 V. Barone and M. Cossi, *J. Phys. Chem. A*, 1998, **102**, 1995–2001.
